# General Screening and Multiple Dissociation Methods for Complementary LC–MS Analysis of Pesticides in Beverages: Potential and Pitfalls

**DOI:** 10.1021/acs.analchem.5c01831

**Published:** 2025-06-27

**Authors:** Romain Giraud, J. C. Yves Le Blanc, Mircea Guna, Gérard Hopfgartner

**Affiliations:** † Life Sciences Mass Spectrometry, Department of Inorganic and Analytical Chemistry, 27212 University of Geneva, 24 Quai Ernest Ansermet, Geneva 4 CH-1205, Switzerland; ‡ SCIEX, Toronto, Ontario M4B 1G2, Canada

## Abstract

A commercial QqTOF platform (ZenoTOF 7600 system) was modified to enable three fragmentation modes: collision-induced dissociation (CID), electron-activated dissociation (EAD), and ultraviolet photodissociation (UVPD) at 266 nm. 168 pesticides, which showed fragmentation in CID, also provide EAD spectra. In the case of UVPD, 158 compounds fragmented under 266 nm photon irradiation. The performance of the novel platform was evaluated using data-independent SWATH CID acquisition for the general screening, and multiple product ion acquisitions were scheduled with CID/EAD/UVPD for confirmatory analysis of pesticides in juice and white and red wine samples. A column-switching liquid chromatography (LC) method with online dilution was developed to inject large volumes (80 μL) into the system. The approach enabled the detection and concentration estimation of approximately 30 pesticides in juices and wines, including insecticides, neonicotinoids, and fungicides. Pesticide limits of detection (LODs) were found to be in the picogram per milliliter to nanogram per milliliter range for MS1 and MS2 acquisitions.

## Graphical abstract

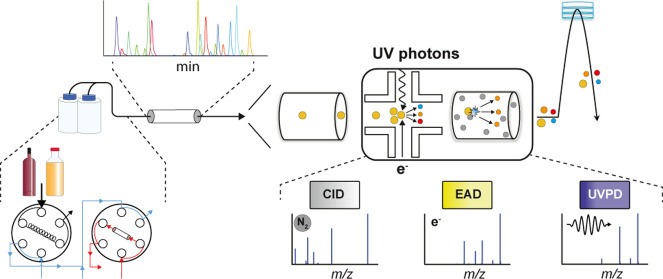

## Introduction

Pesticides are substances that are extensively applied in agriculture to control pests, diseases, and weeds. By safeguarding crops, these compounds enhance production yields and profitability for farmers. However, the various methods of pesticide application result in both direct and indirect exposures, leading to adverse effects on human health and the environment. Neonicotinoids, a relatively new class of pesticides on the market, have become the most widely used insecticides worldwide.^1^ Their prevalence highlights the critical need for regulatory oversight. They became the target of authorities for one major environmental issue, as the origin of the colony collapse disorder (CCD) in honeybees, first reported in 2006,^2^ and for invertebrate aquatic species.^3^ These compounds act as nicotinic acetylcholine receptor (nAChRs) agonists. They bind to the receptors on a postsynaptic membrane with a high affinity, leading to neural hyperactivation and consequential death of the insect.^4^ In addition to the environmental aspect, neonicotinoids’ impact on human health has been recently studied and demonstrated to be involved in breast cancer development by overexpressing the aromatase enzyme (CYP19).^5^

Pesticides can remain on the surface of plants and penetrate plant tissues, subsequently appearing in fruits and vegetables. These pesticides can then be found in processed products, such as wine and juice. Both are widely consumed beverages in many countries, with juices being consumed in large quantities by children and wine by adults. Therefore, to ensure consumer protection, food safety authorities have established legal limit levels, known as maximum residue levels (MRLs), for pesticides and sometimes their metabolites in or on the food.^6^ To note is that most of the time, MRLs in processed products are identical to those of the main product. The regulation does not always consider the fate of the pesticides over the process, where most of the pesticide’s residues significantly decrease during, for example, the winemaking process.^7^ Pesticide processing factors for vinification^8^ and for apple juice^9^ have been published, and in some cases, the literature reports that pesticides completely pass into the wine.^10^

Sample preparation is an important step in liquid chromatography–mass spectrometry (LC–MS) analysis of juices and wines due to significant matrix effects and the presence of isobaric interferences.^11^ Various approaches have been described, including^12^ the quick, easy, cheap, effective, rugged, and safe (QuEChERS) method; liquid–liquid extraction (LLE); solid-phase extraction (SPE); and solid-phase microextraction (SPME). Well-established procedures such as QuEChERS are time-consuming, error-prone, and tedious to automate and generate significant waste. Column-switching or online SPE,^13^ which was primarily developed for bioanalysis, offers an attractive alternative for the automated analysis of liquid samples and has been successfully applied for the determination of pesticides in surface waters,^14^ drinking and ground waters,^15^ wines,^16^ and juices.^17^ Berset et al.^18^ described the analysis of pesticides in wine samples, including neonicotinoids and pesticide metabolites, using direct large volume injection (DI-LVI). Sample dilution of the wine with water was mandatory to avoid analyte loss and maintain a good chromatographic peak shape.

Tandem mass spectrometry is primarily used for quantitative and qualitative analysis by applying collision-induced dissociation (CID). However, CID is nonspecific and highly dependent on the analyte, and collision energy often produces a limited number of product ion fragments, which can impede compound identification and reduce the limit of quantification, especially in the presence of isobaric interferences or high background.^19^

To address these challenges, ultraviolet photodissociation (UVPD) and electron-induced dissociation (ExD), also referenced as electron-induced dissociation (EID) or electron-activated dissociation (EAD), have been described as complementary fragmentation techniques.^20^ UVPD, which uses high-energy photons at different wavelengths (e.g., 213 and 266 nm) and EAD electrons with different kinetic energies (0.1 to 25 eV),^21^ can offer alternative fragmentation pathways and informative spectra. The benefits of UVPD and ExD have been largely reported in proteomics but to a limited extent in low-molecular-weight compounds. Panse et al.^22^ show the benefits of UVPD at 213 nm with complementary fragments for organic micropollutants compared to HCD.

Giraud et al.^23^ compared UVPD, with a 266 nm laser, to CID for a mix of 90 low-molecular-weight compounds, including peptides, pesticides, pharmaceuticals, metabolites, and drugs of abuse. For both activation techniques, complementary fragments and common fragments were observed for most analytes investigated with similar sensitivities.

A comparison was conducted between electron-induced dissociation (EID) at 17–18 eV and collision-induced dissociation (CID) for analyzing a series of agrochemicals using a FT-MS instrument.^24^ EID fragmentation produced a greater variety of fragment ions and complementary ion pairs, leading to a more complete functional group characterization than CID. Ducati et al.^25^ explored the use of a chimeric collision cell integrated into a quadrupole time-of-flight platform. Their study focused on evaluating two dissociation techniques, collision-induced dissociation (CID) and electron-induced dissociation (EAD), for the LC–MS analysis of low-molecular-weight compounds. Compared to CID, EID fragmentation of the species (10 to 20 eV) from standard compounds resulted in additional fragments, primarily due to neutral losses and, in some cases, due to ring openings. In addition, the EAD fragmentation of [M + Na]^+^ or [M + K]^+^ precursor ions showed radical cation and electron impact-type fragments, providing the opportunity to use EI libraries to support analyte identification.

The current work describes a modified QqTOF platform, where ultraviolet photodissociation at 266 nm was added to a chimeric commercial collision cell suitable for collision-induced dissociation and electron-activated dissociation. The system’s performance was investigated for the analysis of pesticides using all three activation methods in a single LC–MS/MS analysis with the aim of additional structural confirmation. A workflow was developed for the screening of 168 pesticides in juices and wines, applying first an untargeted data-independent acquisition LC-SWATH MS screening (retention time, isotopic matching, accurate mass, and MS/MS spectrum), followed by a confirmatory analysis based on targeted scheduled MRM HR with CID, EAD, and UVPD MS/MS spectra. An in-house MS/MS library was built with EAD and UVPD spectra for the identification. A column-switching setup with online dilution was implemented to allow for direct injection of juice and wine samples. The workflow applied was used as a proof of concept for pesticide screening in 37 white and red wines and 21 fruit and vegetable juices. Finally, the concentrations and the limits of detection were estimated in the wine samples.

## Experimental Section

### Chemicals

The standard kit of pesticides, iDQuant, was obtained from Sciex (AB Sciex, Concord, ON, Canada).^26^ A stock solution of iDQuant pesticide standard mix was provided at 10 μg/mL in acetonitrile and stored at −20°C. Thiacloprid, acetamiprid, imidacloprid, and nitenpyram were from Brunschwig (Basel, Switzerland); thiamethoxam was from LabForce AG (Muttenz, Switzerland); dinotefuran was from LubioScience (Zurich, Switzerland); clothianidin was from Fisher Scientific AG (Reinach, Switzerland); and deuterated internal standards thiacloprid d4, clothianidin d3, and thiamethoxam d3 were from Merck (Buchs, Switzerland). Stock solutions of each standard and internal standard were prepared in methanol at 1 mg/mL and kept at −20 °C.

Water (UHPLC-MS grade) was obtained from Huberlab (Aesch, Switzerland), methanol (HPLC grade) from Fischer Scientific AG (Reinach, Switzerland), and ammonium formate from Honeywell (Seelze, Germany).

To conduct the study of pesticide screening in beverages, various juices and wines were collected (Tables S2 and S3). Among the 37 wines acquired, 21 were white wines and 16 were red wines, originating from seven countries (France, Portugal, Italy, Spain, Chile, USA, and Georgia). Twenty-one fruit and vegetable juices were collected, with most of the fruits or vegetables sourced from Europe. These juices included pure orange, apple, pomegranate, grape, grapefruit, pineapple, carrot, and tomato juices, as well as multifruit blends. Wine and juice aliquots (2 mL) were prepared and stored at −20 °C prior to analysis.

### Concentration and Lower Limit of Detection Estimation in Red and White Wines

The samples were centrifuged at 14,000 rpm for 10 min under 4 °C (Megafuge 1.0R, Heraeus Instrument, Schaffhausen, Switzerland). To 247.5 μL of sample, 2.5 μL of internal standards—thiacloprid d4, clothianidin d3, and thiamethoxam d3 at 10 μg/mL—were spiked at a final concentration of 100 ng/mL and vortexed for 30 s. For the estimation of concentration in wine samples, the iDQuant mix (1 μg/mL) was spiked into red and white wines (W1, W3, W5, W6, W18, and W37, Table S3), tested free of pesticides, at a final concentration of 1, 50, and 100 ng/mL. LOD was defined as a signal/noise ratio of 3.

### Liquid Chromatography—Column-Switching

The chromatographic system consisted of three LC pumps (Shimadzu, Kyoto, Japan): two LC-10AD HPLC pumps, one for analyte trapping, featuring an FCV-10AL solvent selector, and the second for online dilution. The third pump for chromatography was an LC-30AD UHPLC pump equipped with a low-pressure gradient unit. The samples were injected with an HTC PAL autosampler (CTC Analytics, Zwingen, Switzerland) equipped with a 0.5 mL syringe and a 0.2 mL injection loop and two switching valves (a Vici 10-port and a Rheodyne 6-port) (Figures S1 and S2). The following trapping columns (all from Dr. Maisch, Germany) were investigated: ReproSil-Pur 120 C18-AQ trap column (20 mm length, 4 mm I.D., 5 μm particle size), ReproSil 80 SCX, Reprospher SCX/C8, Repromer 100 SCX, Repropart 80 SCX, and Reprospher 100 C18/WCX (20 mm length, 4 mm I.D., 10 μm particle size). The mobile phase for trapping was 5 mM NH_4_HCO_2_ in H_2_O at a flow rate of 0.1 mL/min, and for washing, 5 mM NH_4_HCO_2_ in MeOH (0.4 mL/min). For online dilution, the mobile phase was 5 mM NH_4_HCO_2_ in H_2_O, and the flow rate was 1 mL/min. The analytes were separated on a reverse-phase Luna C18 column (100 mm length, 2 mm I.D., 2.5 μm particle size, Phenomenex, Switzerland) safeguarded by a C18 guard column (Phenomenex, Switzerland) at 40 °C. Mobile phase A was 5 mM NH_4_HCO_2_ in H_2_O, and mobile phase B was 5 mM NH_4_HCO_2_ in MeOH. For the investigation of different trapping columns, the following gradient was used: starting at 5% B for 1.30 min and increasing to 95% B in 4.7 min, holding for half a minute, and decreasing back to 5% in 0.3 min to end in the re-equilibration step for 4.2 min. For the analysis of wines and juices, the gradient started at 5% B for 1.30 min and increased to 95% B in 13.7 min and held for 1 min to finally decrease to 5% in 0.2 min for re-equilibration for an additional 3.8 min.

Liquid chromatography–mass spectrometry using multimodal dissociation methods—collision-induced dissociation, electron-activated dissociation, and ultraviolet photodissociation

Liquid chromatography was coupled to a modified ZenoTOF 7600 instrument (SCIEX, Concord, ON, Canada) for analyte detection. Ionization in positive mode was achieved using the electrospray OptiFlow Turbo V ion source (Sciex) with a high-flow microelectrode, 50–200 μL/min. The source parameters were set as follows: ion spray voltage (SV): 5500 V; ion source gas 1 and 2 (GS1 and GS2): 40 and 60 psi, respectively; curtain gas: 35 psi; CAD gas: 7 psi; declustering potential (DP): 70 V; and temperature: 400 °C. To implement UVPD fragmentation on the ZenoTOF 7600 system, the EAD cell was modified, and one of the two EAD filaments was replaced by a mirror (12.7 mm diameter—45° angle) to redirect the UV photon beam through a 12.7 mm diameter UV–visible-grade calcium fluoride (CaF_2_) window into the center of the EAD cell. To get the photons from the laser inside the vacuum chamber, a high-transmission UV–visible-grade calcium fluoride (CaF_2_) window was mounted on the vacuum chamber cover (outside diameter, 25.4 mm; 2 mm thickness). The laser used was a 266 nm Nd/YaG laser (Teem Photonics S.A., Meylan, France) with a power of approximately 0.5 μJ (4.7 eV per photon) and pulsing at a frequency of 19 kHz. EAD and UVPD fragmentation techniques were operated in two different modes: the flow-through mode or the simultaneous trapping mode. These two modes were first described by Takashi Baba et al.^21^ on a Q-TOF instrument. In the simultaneous trapping mode, a potential is applied at the exit of the EAD cell to trap precursor ions within the cell while they are irradiated with UV photons or electrons. In the case of the flow-through mode, the process occurs in two steps. First, precursor ions are trapped by applying a potential at the exit of the EAD cell and accumulating a certain number of ions. Then, in the second step, another potential is applied at the entrance of the EAD cell to trap the ions without allowing new ones to enter, and UV photons or electrons subsequently irradiate them. This work simultaneous trapping mode was applied for both EAD and UVPD experiments. Product ion spectra of standards were acquired for each activation technique by LC–MS under the following conditions: (i) CID, the collision energy range was from 10 to 100 eV with steps of 10 eV; (ii) EAD, the electron kinetic energy range was from 0 to 25 eV with steps of 2 eV; and (iii) UVPD, the reaction time (irradiation time) was in the range of 60 ms.

Pesticide analysis data were acquired following two acquisition methods: (1) SWATH—CID and (2) scheduled MRM HR—CID/EAD/UVPD on a research-grade version of Sciex OS 3.0. For both acquisition modes, a TOF MS experiment with 90 ms accumulation time was set, and Zeno pulsing was applied for MS/MS. For the SWATH acquisition, Q1 windows were set as follows: 100 *m*/*z* between 50 and 150 *m*/*z*, 25 *m*/*z* between 150 and 750 *m*/*z*, and finally, a window of 250 *m*/*z* between 750 and 1000 *m*/*z*. For all experiments, the accumulation time was set to 25 ms. For the MRM HR acquisition, the experiment was scheduled according to the retention time of the pesticides. CID, EAD, and UVPD spectra were acquired sequentially with the MRM HR method in one injection. To ensure a reasonable cycle time on an LC time scale, the CID experiment accumulation time was set at 15 to 35 ms, and the EAD and UVPD experiments accumulation time was set at 60 to 90 ms with a reaction time of 20 to 25 ms, depending on the need. Collision energy (CE) for CID in SWATH acquisition and MRM HR acquisition was set at 35 eV with a collision energy spread (CES) of 20 eV. The kinetic energy (KE) of the electrons was 18 eV with an electron beam current of 3500 nA and EAD RF at 150 Da for both UVPD and EAD. For EAD and UVPD experiments, Q2 was operated at 10 eV.

LC–MS/MS acquisitions were processed with SCIEX OS software (SCIEX, version 3.0) using Explorer and Analytics for qualitative and quantitative data analysis. Low smoothing was used in the processing data, interference resolution was set at 50%, and two SCIEX libraries (Metabolite HR-MS/MS and Pesticides HR-MS/MS spectral) were used. Confidence levels for CID SWATH acquisition were <5 ppm for acceptable and 5–10 ppm for marginal difference, error retention time inferior to 2.5% for acceptable and 40% for marginal difference, isotope ratio inferior to 5% for acceptable and 20% for marginal difference, and finally, library hit score 80% for acceptable and 65% for marginal difference.

## Results and Discussion

Liquid Chromatography Coupled to Tandem Mass Spectrometry—CID, EAD, and UVPD Fragmentation

### Comparison of CID, EAD, and UVPD

For targeted screening of pesticides by LC–MS, collision-induced dissociation is the most widely used activation technique on low-resolution (MRM) or high-resolution (DDA/DIA) mass spectrometers. Commercial or in-house libraries are applied for confirmation or to build MRM methods. However, CID shows limitations with regard to the number and specificity of the fragments for a given analyte, which depends on the collision energy. This can challenge the unambiguous identification of known analytes or the characterization of unknowns. The use of orthogonal activation techniques, such as electron activation dissociation or ultraviolet photodissociation, in the same LC run for additional structural information would therefore be of interest. UVPD, on the other hand, is based on photon absorption at chromophores, causing fragmentation near the absorption site, direct dissociation, or at weaker points along the molecule via internal vibrational redistribution.^27^ However, these mechanisms can lead to diverse fragments.

To implement UVPD fragmentation on the ZenoTOF 7600 system, the EAD cell was modified and one of the two EAD filaments was replaced by a mirror to redirect the UV photon beam in the center of the EAD cell, as shown in Figure 1. One high-transmission UV–visible-grade calcium fluoride (CaF_2_) window was mounted on the vacuum chamber cover to get the photons from the laser inside the vacuum chamber. The software was adapted to add UVPD acquisition functionalities in addition to CID and EAD by using DDA, DIA, and MRM HR acquisition modes.

**Figure 1:**
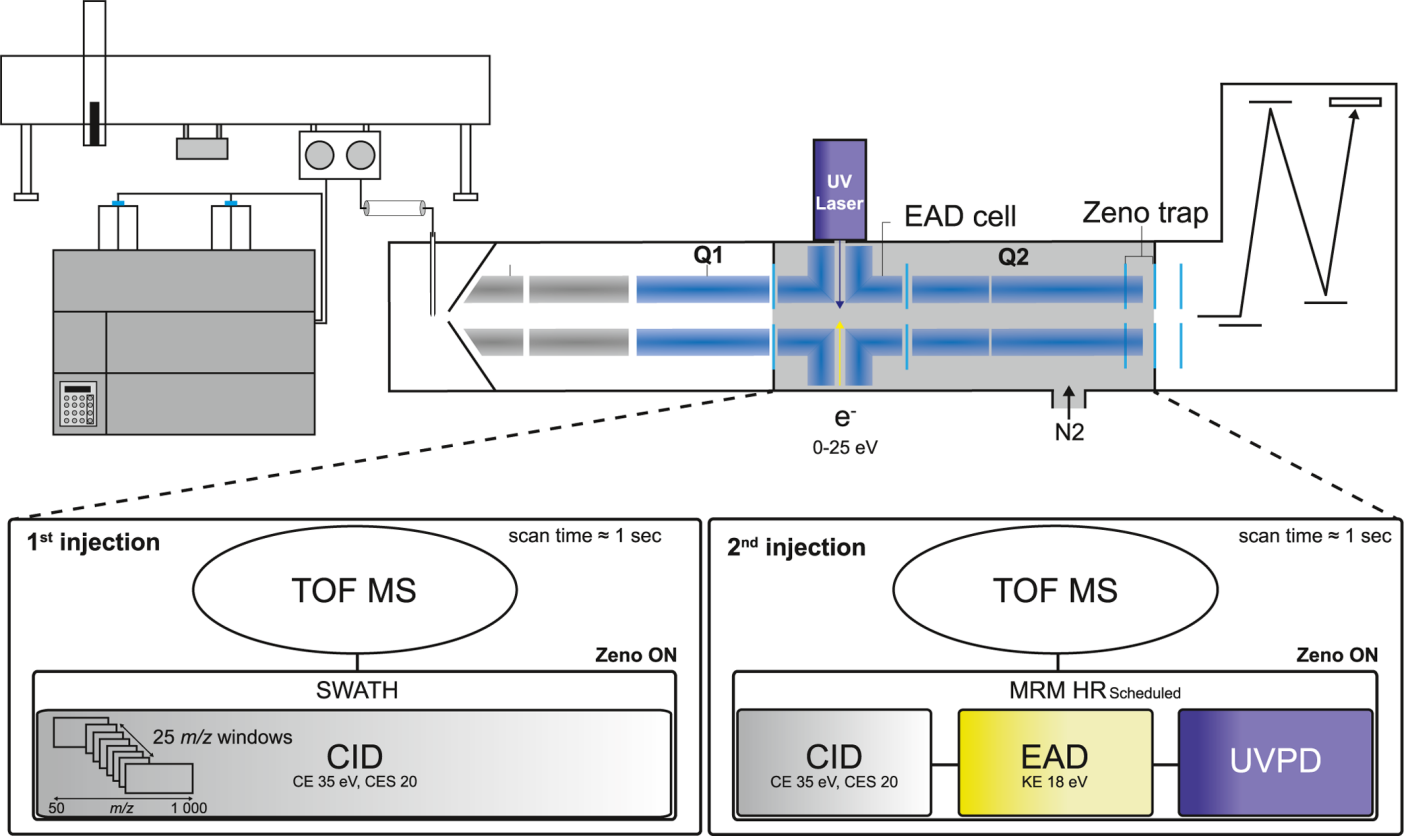
Schematic of a modified ZenoTOF 7600 with the implementation of UVPD and LC–MS/MS workflow: 1st injection in data-independent acquisition (DIA) with SWATH CID (35 ms accumulation time) and 2nd injection with scheduled MRM HR CID (25 ms accumulation time) and EAD and UVPD (90 ms accumulation time and 25 ms reaction time).

To investigate the performance of all three activation methods and to build specific HR-MS/MS libraries, multiple product ion spectra, with precursor isolation at unit mass, were recorded in positive mode for 168 pesticide standards by LC–MS (Table S1). CID and EAD spectra with fragments could be obtained for all 168 pesticides, while for UVPD, 158 spectra showed fragments. All three techniques showed similar and different odd and even electron fragments and differences too. It is reported by our group, based on the pesticide investigation, that fragmentation behavior in UVPD and EAD is closely linked to molecular structure and can be predicted using simple cheminformatics models. The formation of [M + H]^2+•^radicals in EAD correlates with their thermodynamic stability, supporting an electron removal component in the EAD mechanism. These findings demonstrate that even basic modeling approaches can guide the rational application of advanced MS/MS techniques and offer valuable insights into complex microscopic activation mechanisms.^28^

Doubly charged radical cations were observed for 18 analytes and only for EAD activation (Table S1 and Figures S3–S4). Preliminary investigations were performed using a 213 nm laser (20 μJ, 1 kHz). The same setup was applied as for the 266 nm laser with just changing the laser. The UVPD spectra of boscalid and dimethomorph at 213 nm show a significant increase in the number of fragments produced and their intensity compared to 266 nm (Figures S5 and S6). The use of lasers may be of interest with regard to observed fragments as well as to tune the fragmentation selectivity of coeluting compounds.

### LC–MS/MS General Screening Workflows

In addition to data-dependent acquisition (DDA), data-independent acquisition (DIA) LC–MS workflows such as SWATH MS using CID on fast-acquiring QqTOF platforms have gained interest for untargeted screening. The strategy applied (Figure 1) is to perform a general screening in a first LC–MS/MS analysis using DIA SWATH MS with CID to detect known analytes and unknowns such as metabolites or contaminants. In a second step, in addition to a full scan acquisition, a targeted LC–MS/MS analysis is performed using scheduled MRM HR CID/EAD/UVPD using all three fragmentation methods in less than 1 s without compromising LC resolution.^29^ The TOF MS-Zeno trapping scheduled MRM HR method is illustrated in Figure 2. Each block unit incorporates CID, EAD, and UVPD MS/MS and the TOF MS spectra for each analyte. This setup allows four MRM HR CID/EAD/UVPD blocks to overlap, each with an accumulation time of 235 ms. Bridging targeted MRM HR and untargeted SWATH LC–MS has been described on the ZenoTOF in a single run but only with CID.^30^ One could certainly consider the same approach with CID/EAD/UVPD, but this is currently challenging to implement on an LC time scale.

**Figure 2:**
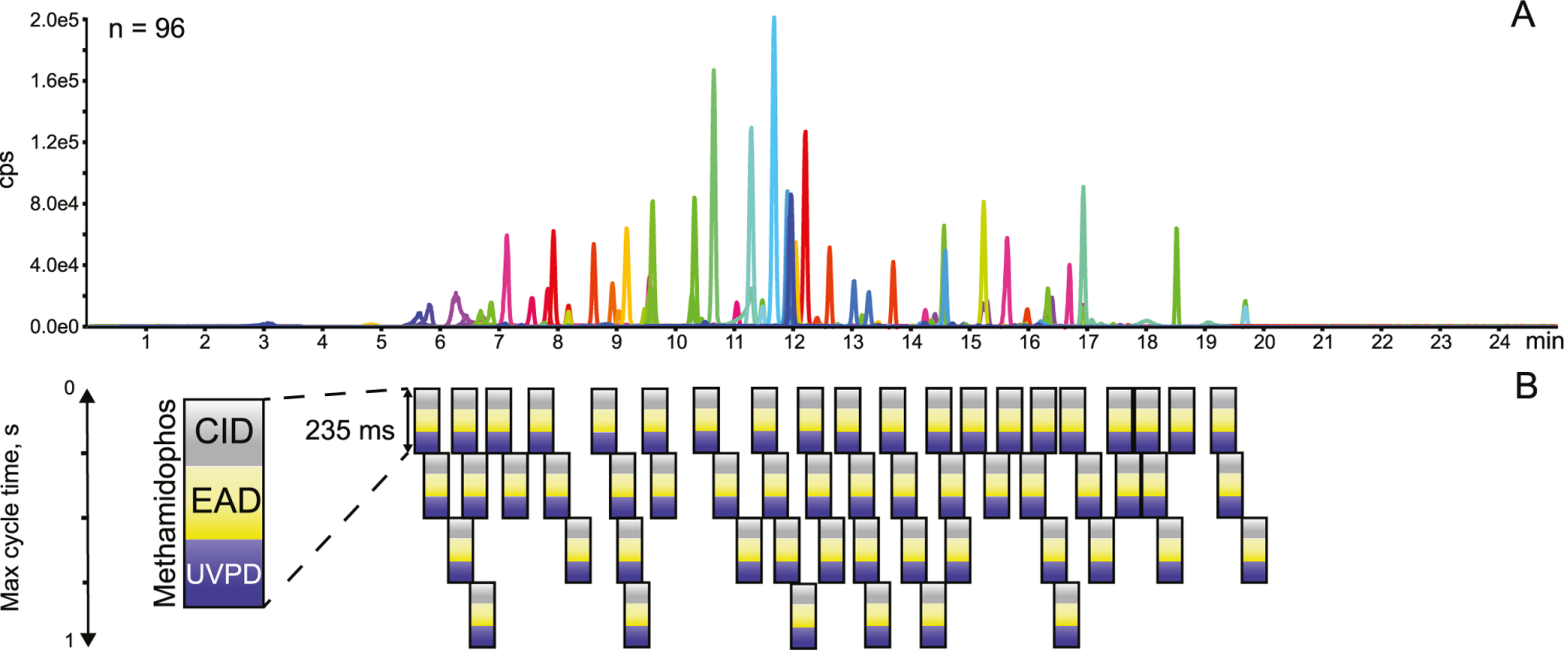
(A) MS 1 extracted ion current of 96 pesticides and (B) scheduled MRM HR CID/EAD/UVPD acquisition scheme.

The application of Zeno trapping shows an approximate gain in sensitivity of five times, and about 100 analytes can be monitored in a single LC–MS run. In addition to providing better signals for the product ion spectra, the information gained with EAD and UVPD can be used to confirm the general screening results or provide additional structural information. All three modes are also suitable for quantitative analysis. The benefit of the additional activation methods is a different fragmentation selectivity toward coeluting isobaric compounds or background signals for a given analyte. The MS response is analyte-dependent, and the limit of detection (LOD) based on standard solutions was estimated to be in the range of pg/mL to ng/mL based on an injection volume of 80 μL (S/*N* > 3). A more detailed evaluation of LOD for detected analysis is presented in the section on wine sample analysis.

LC–MS Column Switching with Online Dilution for the Direct Analysis of Wines and Juices

QuEChERS is one of the most popular sample preparation approaches for the analysis of food samples. Still, the procedure is quite complex with limited sample throughput and generates a significant amount of waste. Direct injection of diluted juice or wine samples onto an LC column is possible but at the cost of sensitivity and assay robustness. Column switching is primarily used in bioanalysis for the direct injection of plasma and urine and is also suitable for the analysis of food liquids. Several cartridges were evaluated under various trapping conditions to determine the most suitable parameters for pesticide retention. Seven pesticides were selected for this purpose: acetamiprid, clothianidin, dinotefuran, imidacloprid, nitenpyram, thiacloprid, and thiamethoxam (Figure S7). Juices are mostly aqueous, while wine samples can contain up to 15% ethanol, affecting retention of polar analytes. Trapping tests were conducted using standards dissolved in H_2_O/EtOH (85/15, v/v). Based on the predicted analyte charge state (Figure S8), two different trapping mobile phases were tested, including 5 mM ammonium formate (pH 6.2) and 1% formic acid (pH 2.1). Among the four trap columns tested, ReproSil-Pur 120 C18-AQ and Reprospher 100 C18/WCX provided the best results in the number of analytes retained and the signal intensities (Figure S9). In contrast, the ReproSil 80 SCX performed poorly, with no trapping of dinotefuran, clothianidin, and thiamethoxam under both buffer conditions. Additionally, using 1% formic acid in the sample led to a loss of nitenpyram when using strong cation exchange or mixed-mode trapping columns (Figure S10). Ultimately, ReproSil-Pur 120 C18-AQ proved to be the most effective trapping column under both buffer conditions, in particular for nitenpyram (Figure S9).

Online dilution was implemented before the trapping column to reduce the elution strength of wine samples containing ethanol. The injection flow rate was set to 0.1 mL/min, and the dilution flow rate ranged between 0.1 and 1 mL/min, resulting in a dilution ratio from 2 to 11 without loss of analytes (Figure S11). The trapping time was set to 1 min, and the largest washing volume of 1.1 mL allowed the removal of polar compounds (Figure S12A). The online dilution allows for the refocusing of early eluting analytes, such as nitenpyram (Figure S12B).

### LC–MS/MS Analysis of Fruit and Vegetable Juices

Pesticide residue analysis was conducted on 21 fruits and vegetable juices. Across all juice samples, twenty-one different pesticides, mostly fungicides, were detected and confirmed, with different frequencies. A summary is presented in Table S2, and their occurrence is shown in Figure S13, where pyrimethanil, boscalid, metalaxyl, tebuconazole, and carbendazim occur in at least five samples. As a note, two neonicotinoids, acetamiprid and imidacloprid, controversial pesticides with country-specific regulations, were found four and one times, respectively, in apple juices. Boscalid, a commonly detected fungicide, was found in carrot juice, as well as in six other juice samples. The presence of boscalid was screened in apple juice J9 by generating the XIC at *m*/*z* 343.039 (Figure 3A). From the three peaks observed, only RT = 15.08 min fit the retention time of the standard.

**Figure 3:**
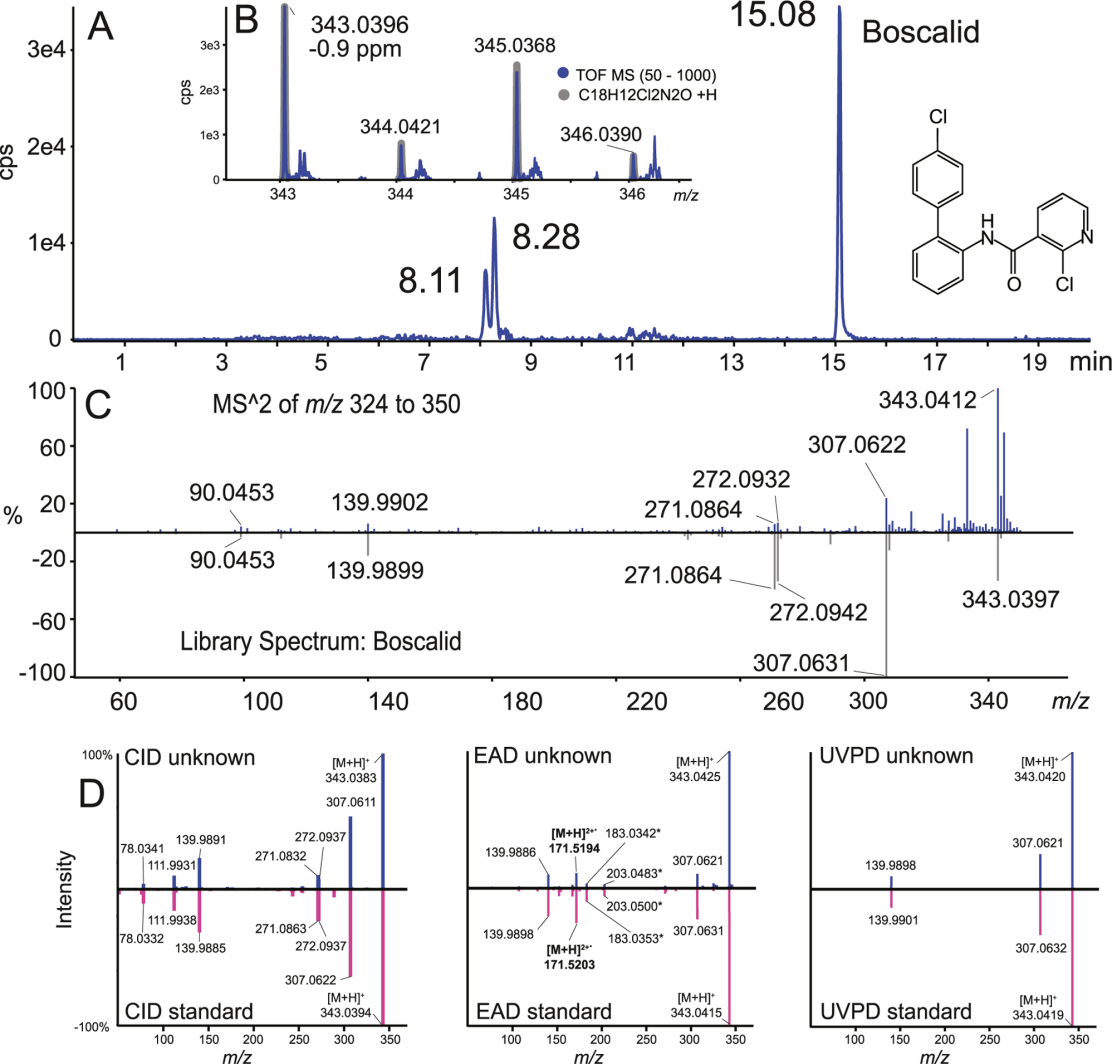
LC–MS analysis of apple juice J9. (A) TOF MS XIC of boscalid (C_18_H_12_Cl_2_N_2_O) *m*/*z* 343.039; (B) isotope pattern matching: gray—theoretical and blue—experimental; (C) SWATH CID library search of the peak at 15.08 min: experimental (top) and library hit with boscalid (bottom); (D) CID, EAD, and UVPD MRM HR—apple juice peak at RT = 15.08 min (top) and spectra of boscalid standard (bottom).

The calculated isotopic distribution is compared to the experimental one (accuracy on the exact mass of −0.9 ppm) as well as the SWATH experimental spectrum (Figure 3B,C) with the one from the library (reverse fit scoring).

In the second injection, the targeted scheduled MRM HR mode using multiple activation methods (CID, EAD, and UVPD) allows further confirmation of the identification of boscalid, as shown in Figure 3D. With unit mass isolation and Zeno trapping, the *S*/*N* of the product ion spectra is significantly increased and therefore the limit of detection. The CID and the UVPD spectra show different fragment patterns as UVPD activation depends on the analyte chromophore, which is more specific than CID, leading to a selective fragmentation pathway. The EAD spectrum shows a unique fragment ion at *m*/*z* 171.5181, corresponding to a doubly charged radical cation [M + H]^2+.^. All three characteristic spectra, which could be acquired in the same LC–MS analysis, allow us to confirm the identity of boscalid and minimize the risk of false positives, which could occur with a single spectrum. Furthermore, quantification would be possible with MRMHR and Zeno trapping, with all three methods on the same or different fragments with improved *S*/*N* and selectivity. Representative MS/MS spectra for the detected pesticides in juices can be found in Figures S14–S31, which clearly supports the benefit of the workflow for complementary identification.

### LC–MS/MS Analysis of Red and White Wines

Pesticide analysis was conducted on 21 white and 16 red wines (Table S3). Wine samples required online dilution for efficient trapping of analytes on the C18-AQ trap column. Twenty-seven pesticides were detected (Figure S32), and the concentrations were estimated using stable isotope-labeled internal standard single-point calibration (Table S4). About 70% of the pesticides found were fungicides, and 30% were insecticides, including neonicotinoids like acetamiprid and imidacloprid. In many cases for wines, scheduled MRM HR experiments were necessary to confirm identification, as the quality of the SWATH acquisition spectrum, even with Zeno pulsing, was limited. MRM HR with CID, as well as EAD, further confirms that the analyte detected was the correct one. Representative MS/MS spectra for the detected pesticides in wines can be found in Figures S33–S42.

Dimethomorph, a commonly used fungicide in grape cultivation against mildew, was detected in almost all the wines except for two, one of which was produced in 1991 before the commercialization of dimethomorph. Figure 4A shows the TOF MS XICs of imidacloprid at RT 8.88 min and dimethomorph at RT 14.7 min detected in red wine, sample W35. Confirmation with MS/MS spectra from standards was performed with all three activation methods, CID, EAD, and UVPD, as illustrated in Figures 4B–D and 4E–G for imidacloprid and dimethomorph, respectively. Boscalid MS/MS product ion spectra show a [M + H]^2+.^ ion at *m*/*z* = 194.0655 (Figure 4F).

**Figure 4:**
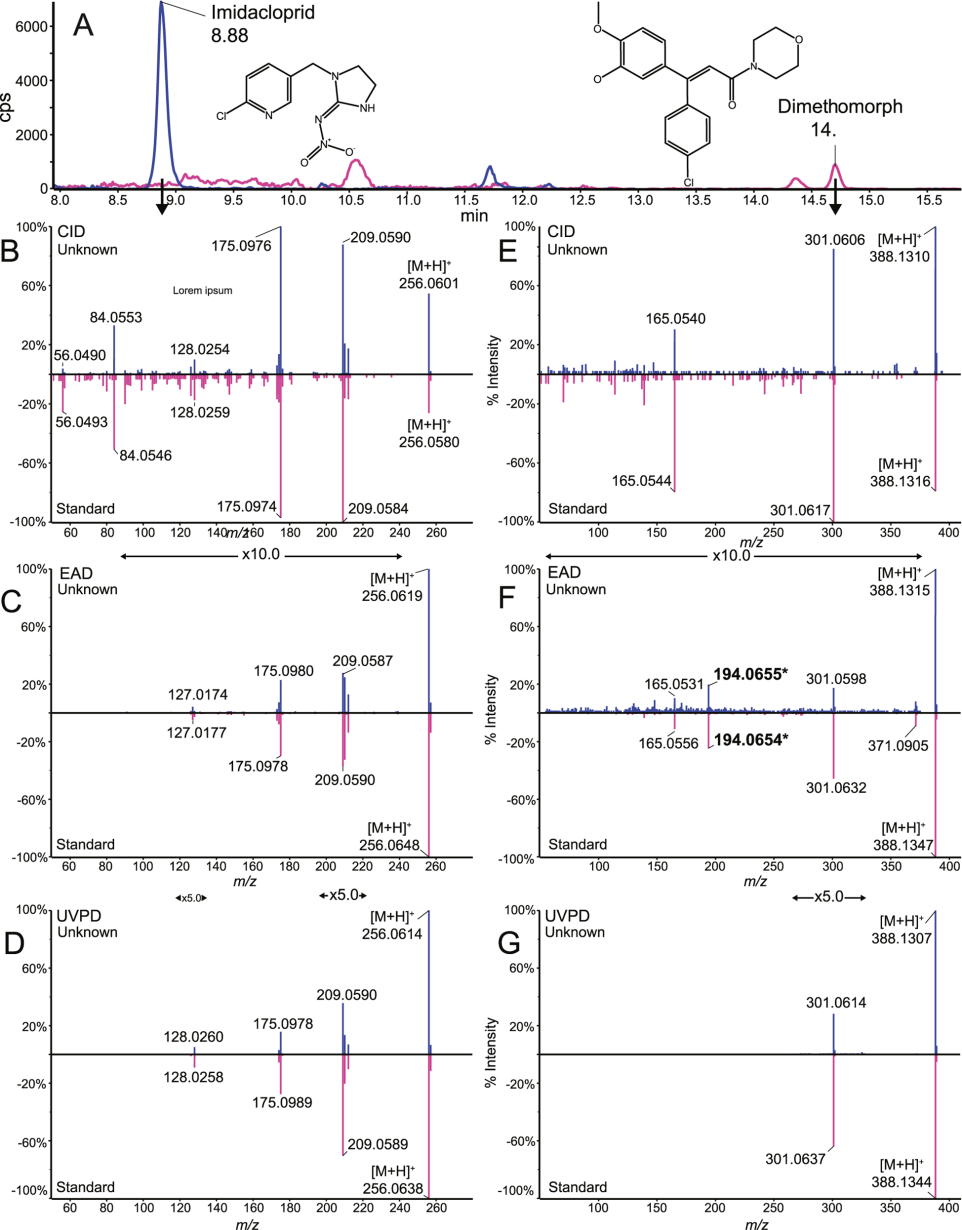
LC–MS analysis of the wine sample (W35). TOF MS XIC of imidacloprid and dimethomorph detected in an Asian red wine (A), with their fragmentation spectra obtained using CID (B,E), EAD (C,F), and UVPD (D,G) in scheduled Zeno MRM HR mode. The doubly charged radical cation is labeled in bold with an asterisk.

To characterize the quantification performance of the approach, the concentration levels in wine samples were estimated using the single-point calibration of XIC of the TOF experiment using intensity ratios of analyte/IS of standards spiked in pesticide-free red and white wine. Using an 80 μL injection volume of sample, the estimated concentration from the 27 pesticides detected ranged from 75.6 to 35.4 ng/mL (Table S4). For neonicotinoids, the estimated concentrations were 2.8 ng/mL for acetamiprid and 12.3 ng/mL for imidacloprid. LOD values were calculated for all pesticides using TOF MS signal intensities and CID, EAD, and UVPD single fragments (Table S5). The results show that TOF MS and TOF MS/MS exhibit similar LOD down to the pg/mL level but are compound-dependent. For boscalid, the CID LOD was higher than for EAD or UVPD, showing the selectivity and sensitivity benefits of alternative fragmentation techniques.

## Conclusions

A commercial QqTOF platform, the ZenoTOF 7600 system from SCIEX, was modified to add UVPD to CID and EAD fragmentation. Product ion spectra could be acquired for 168 pesticides in CID and EAD and 156 pesticides in UVPD at 266 nm. UVPD provided additional spectral information complementary to CID and EAD, which can be used for confirmatory analysis. Quantitative analysis could also benefit from different activation techniques with regard to selectivity, as fragmentation of coeluting isobaric interferences would behave differently. A 213 nm more energetic laser has shown better fragmentation, and its application is under investigation. With the current software features, LC–MS analyses can be performed either with DDA or DIA using a single/dual activation method or in product ion mode (MRM HR) with all three activation techniques in the same acquisition with or without Zeno trapping. As proof of concept, the novel platform’s performance and application were investigated with the screening of pesticides in juice and wine samples. DIA SWATH acquisition using CID was found to be a versatile approach for general screening where each analysis can be reinterrogated at any time, while the use of all three acquisition activation methods in a single LC–MS improves the identification of the analytes. With the scheduled MRM HR mode, about 100 different precursors can be considered in a single analysis for confirmatory analysis or additional structural information. Wine and juice samples were analyzed directly without any sample preparation using column switching and online dilution for wine samples. Most detected pesticides were fungicides (around 70%), with the remainder being insecticides. Ultimately, LOD values were determined for detected pesticides in white and red wine samples, reaching the picogram per milliliter to nanogram per milliliter range using an injection volume of 80 μL for both TOF MS and TOF MS/MS levels using the three activation methods. This highlights the advantage of selecting the most suitable method for optimal sensitivity. The approach described could also be beneficial for the general screening of pharmaceuticals and metabolites in plasma and urine or for lipid analysis.

## Supplementary Material



## Notes

List of 168 pesticides; description of fruit, vegetable juices, and wines analyzed; estimated concentrations and LODs measured in wine samples; schematic and settings of column switching; MS/MS spectra of pesticides detected in samples; and CID, EAD, and UVPD mass spectra of the 168 pesticides (PDF)
