# Supplementary material for: General Screening and Multiple Dissociation Methods for Complementary LC–MS Analysis of Pesticides in Beverages: Potential and Pitfalls
Source: Anal Chem. 2025 Jun 27;97(27):14503–11. doi: 10.1021/acs.analchem.5c01831 (PMC12268824; doi:10.1021/acs.analchem.5c01831)
Supplement: Supplementary file 1 [file ac5c01831_si_001.pdf]

Supporting Information

General Screening and Multiple Dissociation Methods for Complementary LC-MS Analysis: Pesticides in Beverages

Romain Giraud[1], J. C. Yves Le Blanc[2], Mircea Guna[2], Gérard Hopfgartner[1]*

[1]*Life Sciences Mass Spectrometry, Department of Inorganic and Analytical Chemistry, University of Geneva, 24 Quai Ernest Ansermet, CH-1205, Geneva 4, Switzerland*
[2]*SCIEX, Toronto, Ontario, Canada*

LC-MS/MS, column switching, pesticides, juice, wine, general screening, CID-EAD-UVPD

ORCID: Romain Giraud: 0009-0006-8180-2874

ORCID: J.C. Yves Le Blanc : 0000-0002-3801-3590

ORCID: Gérard Hopfgartner: 0000-0002-9087-606X

*corresponding author e-mail: gerard.hopfgartner@unige.ch

Table of contents

List of Figures

Figure S1. Schematic of column switching and online dilution scheme across three steps, injection (A), trapping (B), analysis (C). Valve 1 was a six-port valve and for valve 2 either a 6 or a 10-port valve were used. 6

Figure S2. Column switching valves positions and timing along the acquisition. 6

Figure S3. Boscalid product ion fragment (m/z 171.5181) intensity versus kinetic energy. 7

Figure S4. Dimethomorph product ion fragment (m/z 194.0655) intensity versus kinetic energy.... 7

Figure S5. MRM HR UVPD spectra of boscalid at 266 nm (A) and 213 nm (B). 8

Figure S6. MRM HR UVPD spectrum of dimethomorph at 266 nm (A) and 213 nm (B). 8

Figure S7. Structures of the seven neonicotinoids investigated and their pKa and LogP values. 9

Figure S8. Predicted analyte charge states in function of pH (ACD lab Percepta 2012). 9

Figure S9. Signal intensity of seven neonicotinoids for different trap columns, trapping mobile phase 5 mM ammonium formate, injection volume of 40 µL. The analytes were dissolved in a mixture H2O/EtOH 85/15 (v/v) and 5 mM ammonium formate. Reprosil 80 SCX (RSil-80), Repropart 80 SCX (RP-80), Reprosil-Pur 120 C18-AQ (RSil-120), Reprospher 100 C18/WCX (RS-100). 10

Figure S10. Signal intensity of seven neonicotinoids for different trap columns, trapping mobile phase 5 mM ammonium formate, injection volume of 40 µL. The analytes were dissolved in a mixture H2O/EtOH 85/15 (v/v) and 1% formic acid. Reprosil 80 SCX (RSil-80), Repropart 80 SCX (RP-80), Reprosil-Pur 120 C18-AQ (RSil-120), Reprospher 100 C18/WCX (RS-100). 10

Figure S11. Pesticides signal intensities spiked in red wine in function on-line dilution flow rate. Injection flow rate was of 0.1 ml/min. 11

Figure S12. Impact of the washing volume on the TICs (A), and peak shape as observed in the XICs for the analytes nitenpyram, RT 6.4 min (B) and acetamiprid RT 7.0 min (C), injection volume 40 µL. 11

Figure S13. Frequency of pesticides found in juices. 12

Figure S14. Detection of pyrimethanil in grapefruit juice sample J15 A) TOF MS XIC of m/z 280.154. B) Upper trace CID SWATH/MS spectrum of peak at RT = 14.9 and lower trace SWATH MS/MS CID spectrum of pyrimethanil C) MRMHR CID D), MRMHR EAD and E) MRMHR UVPD spectra of peak at RT = 14.9 in sample J16. 12

Figure S15. Detection of metalaxyl in grape juice sample J16 A) TOF MS XIC of m/z 280.154. B) Upper trace CID SWATH/MS spectrum of peak at RT = 13.9 and lower trace SWATH MS/MS CID spectrum of metalaxyl C) MRMHR CID D), MRMHR EAD and E) MRMHR UVPD spectra of peak at RT = 13.9 in sample J16. 13

Figure S16. Detection of tebuconazole in multifruit juice sample J3 A) TOF MS XIC of m/z 308.152. B) Upper trace CID SWATH/MS spectrum of peak at RT = 16.8 and lower trace SWATH MS/MS CID spectrum of tebuconazole C) MRMHR CID D), MRMHR EAD and E) MRMHR UVPD spectra of peak at RT = 16.8 in sample J3. 13

Figure S17. Detection of carbendazim in apple juice J9 A) TOF MS XIC of m/z 192.077. B) Upper trace CID SWATH/MS spectrum of peak at RT = 10.9 and lower trace SWATH MS/MS CID spectrum of carbendazim C) MRMHR CID D), MRMHR EAD and E) MRMHR UVPD spectra of peak at RT = 10.9 in sample J9. 14

Figure S18. Detection of cyprodinil in multifruit juice sample J3 A) TOF MS XIC of m/z 226.134. B) Upper trace CID SWATH/MS spectrum of peak at RT = 16.6 and lower trace SWATH MS/MS CID spectrum of cyprodinil C) MRMHR CID D), MRMHR EAD and E) MRMHR UVPD spectra of peak at RT = 16.6 in sample J3. 14

Figure S19. Detection of imazalil in grapefruit juice sample J15 A) TOF MS XIC of m/z 297.056. B) Upper trace CID SWATH/MS spectrum of peak at RT = 16.5 and lower trace SWATH MS/MS CID spectrum of imazalil C) MRMHR CID D), MRMHR and E) MRMHR UVPD spectra of peak at RT = 16.5 in sample J15.. 15
Figure S20. Detection of azoxystrobin in pomegranate juice sample J7 A) TOF MS XIC of m/z 404.124. B) Upper trace CID SWATH/MS spectrum of peak at RT = 14.7 and lower trace SWATH MS/MS CID spectrum of azoxystrobin C) MRMHR CID D), MRMHR and E) MRMHR UVPD spectra of peak at RT = 14.7 in sample J7. 15
Figure S21. Detection of pyraclostrobin in multifruit juice sample J3 A) TOF MS XIC of m/z 388.106. B) Upper trace CID SWATH/MS spectrum of peak at RT = 16.8 and lower trace SWATH MS/MS CID spectrum of pyraclostrobin C) MRMHR CID D), MRMHR EAD and E) MRMHR UVPD spectra of peak at RT = 16.8 in sample J3. 16
Figure S22. Detection tebufenozide in grape juice sample J16 A) TOF MS XIC of m/z 353.22. B) Upper trace CID SWATH/MS spectrum of peak at RT = 16.3 and lower trace SWATH MS/MS CID spectrum of tebufenozide C) MRMHR CID D), MRMHR EAD and E) MRMHR UVPD spectra of peak at RT = 16.3 in sample J16. 16
Figure S23. Detection of flonicamid in apple juice sample J8 A) TOF MS XIC of m/z 230.054. B) Upper trace CID SWATH/MS spectrum of peak at RT = 8.3 and lower trace SWATH MS/MS CID spectrum of flonicamid C) MRMHR CID D), MRMHR EAD and E) MRMHR UVPD spectra of peak at RT = 8.3 in sample J8. 17
Figure S24. Detection of fenhexamid in tomato juice sample J17 A) TOF MS XIC of m/z 302.071. B) Upper trace CID SWATH/MS spectrum of peak at RT = 15.8 and lower trace SWATH MS/MS CID spectrum of fenhexamid C) MRMHR CID D), MRMHR EAD and E) MRMHR UVPD spectra of peak at RT = 15.8 in sample J17. 17
Figure S25. Detection of mandipropamid in tomato juice sample J17 A) TOF MS XIC of m/z 412.131. B) Upper trace CID SWATH/MS spectrum of peak at RT = 15.1 and lower trace SWATH MS/MS CID spectrum of mandipropamid C) MRMHR CID D), MRMHR EAD, and E) MRMHR UVPD spectra of peak at RT = 15.1 in sample J17. 18
Figure S26. Detection of methoxyfenozide at in grape juice sample J16 A) TOF MS XIC of m/z 369.217 B) Upper trace CID SWATH/MS spectrum of peak at RT = 15.9 and lower trace SWATH MS/MS CID spectrum of methoxyfenozide C) MRMHR spectra CID D), MRMHR EAD, and E) MRMHR UVPD spectra of peak at RT = 17.2 in sample J16. 18
Figure S27. Detection of trifloxystrobin in multifruit juice sample J3 A) TOF MS XIC of m/z 409.137 B) Upper trace CID SWATH/MS spectrum of peak at RT = 17.2 and lower trace SWATH MS/MS CID spectrum of trifloxystrobin C) MRMHR CID D), MRMHR EAD, and E) MRMHR UVPD spectra of peak at RT = 17.2 in sample J3. 19
Figure S28. Detection of chlorantraniliprole in apple juice sample J20 A) TOF MS XIC of m/z 481.978 B) Upper trace CID SWATH/MS spectrum of peak at RT = 14.4 and lower trace SWATH MS/MS CID spectrum of thlorantraniliprole C) MRMHR CID D), MRMHR EAD, and E) MRMHR UVPD spectra of peak at RT = 14.4 in sample J20................. 19
Figure S29. Detection of acetamiprid in apple juice sample J9 A) TOF MS XIC of m/z 223.075 B) Upper trace CID SWATH/MS spectrum of peak at RT = 10.2 and lower trace SWATH MS/MS CID spectrum of acetamiprid C) MRMHR CID D), MRMHR EAD, and E) MRMHR UVPD spectra of peak at RT = 10.2 in sample J9. 20
Figure S30. Detection of tetraconazole in apple juice sample J20 A) TOF MS XIC of m/z 372.029 B) Upper trace CID SWATH/MS spectrum of peak at RT = 15.9 and lower trace SWATH MS/MS CID spectrum of tetraconazole C) MRMHR CID D), MRMHR EAD, and E) MRMHR UVPD spectra of peak at RT = 15.9 in sample J20. 20
Figure S31. Detection of pirimicarb in apple juice sample J2 A) TOF MS XIC of m/z 239.150 B) Upper trace CID SWATH/MS spectrum of peak at RT = 13.5 and lower trace SWATH

MS/MS CID spectrum of pirimicarb C) MRMHR CID D), MRMHR EAD, and E) MRMHR UVPD spectra of peak at RT = 13.5 in sample J2. 21
Figure S32. Frequency of pesticides found in red and white wines.. 21
Figure S33. Detection of spiroxamine in a white wine sample W20 A) TOF MS XIC of m/z 239.150 B) Upper trace CID SWATH/MS spectrum of peak at RT = 16.1 and lower trace SWATH MS/MS CID spectrum of spiroxamine C) MRMHR CID D), MRMHR EAD, and E) MRMHR UVPD spectra of peak at RT = 16.1 in sample W20. 22
Figure S34. Detection of kresoxim-methyl in a white wine sample W31 A) TOF MS XIC of m/z 314.139 B) Upper trace CID SWATH/MS spectrum of peak at RT = 16.4 and lower trace SWATH MS/MS CID spectrum of spiroxamine C) MRMHR CID D), MRMHR EAD, and E) MRMHR UVPD spectra of peak at RT = 16.4 in sample W31......................... 22
Figure S35. Detection of zoxamide in a white wine sample W31 A) TOF MS XIC of m/z 336.032 B) Upper trace CID SWATH/MS spectrum of peak at RT = 16.7 and lower trace SWATH MS/MS CID spectrum of zoxamide C) MRMHR CID D), MRMHR EAD, and E) MRMHR UVPD spectra of peak at RT = 16.7 in sample W31. 23
Figure S36. Detection of benalaxyl in a red wine sample W4 A) TOF MS XIC of m/z 326.175 B) Upper trace CID SWATH/MS spectrum of peak at RT = 16.6 and lower trace SWATH MS/MS CID spectrum of benalaxyl C) MRMHR CID D), MRMHR EAD, and E) MRMHR UVPD spectra of peak at RT = 16.6 in sample W4. 23
Figure S37. Detection of spinosad in a red wine sample W5 A) TOF MS XIC of m/z 732.468 B) Upper trace CID SWATH/MS spectrum of peak at RT = 18.2 and lower trace SWATH MS/MS CID spectrum of spinosad C) MRMHR CID D), MRMHR EAD, and E) MRMHR UVPD spectra of peak at RT = 18.2 in sample W5. 24
Figure S38. Detection of clofentezine in a white wine sample W18 A) TOF MS XIC of m/z 303.020 B) Upper trace CID SWATH/MS spectrum of peak at RT = 16.9 and lower trace SWATH MS/MS CID spectrum of clofentezine C) MRMHR CID D), MRMHR EAD, and E) MRMHR UVPD spectra of peak at RT = 16.9 in sample W18. 24
Figure S39. Detection of buprofezin in a white wine sample W18 A) TOF MS XIC of m/z 306.163 B) Upper trace CID SWATH/MS spectrum of peak at RT = 17.7 and lower trace SWATH MS/MS CID spectrum of buprofezin C) MRMHR CID D), MRMHR EAD, and E) MRMHR UVPD spectra of peak at RT = 17.7 in sample W18. 25
Figure S40. Detection of penconazole in a red wine sample W34 A) TOF MS XIC of m/z 284.072 B) Upper trace CID SWATH/MS spectrum of peak at RT = 16.5 and lower trace SWATH MS/MS CID spectrum of penconazole C) MRMHR CID D), MRMHR EAD, and E) MRMHR UVPD spectra of peak at RT = 16.5 in sample W34. 25
Figure S41. Detection of iprovalicarb in a red wine sample W13 A) TOF MS XIC of m/z 321.217 B) Upper trace CID SWATH/MS spectrum of peak at RT = 15.7 and lower trace SWATH MS/MS CID spectrum of penconazole C) MRMHR CID D), MRMHR EAD, and E) MRMHR UVPD spectra of peak at RT = 15.7 in sample W13. 26
Figure S42. Detection of difenoconazole in red wine sample W17 A) TOF MS XIC of m/z 406.072 B) Upper trace CID SWATH/MS spectrum of peak at RT = 17.1 and lower trace SWATH MS/MS CID spectrum of difenoconazole C) MRMHR CID D), MRMHR EAD, and E) MRMHR UVPD spectra of peak at RT = 17.1 in sample W17. 26

List of Tables

Table S1. List of the 168 pesticides: CAS number, formula, exact mass Log P, retention time and observed fragment in CID/EAD/UVPD. [1] are the pesticides showing doubly charged radical cation. The MS/MS spectra of standard pesticides in MassBank format can be found at: 10.26037/yareta:idzwd42n4feaddt7fydqefavve 27
Table S2. Description of fruit and vegetable juices analysed which were purchased in France and Switzerland. Pesticides found in these juices as well as their occurrences. 33
Table S3. Summary of red and white wines analysed with the pesticides detected and their occurrences. 35
Table S4. Concentration estimation of pesticides found in red and white wines, n.d. not detected. ... 37
Table S5. Summary of estimated lower limit of detection (LOD) for pesticides detected in juice, red, and white wine (n=27) based on the signal intensity of TOF MS, CID, EAD, and UVPD MS/MS, n.f. no fragment... 38

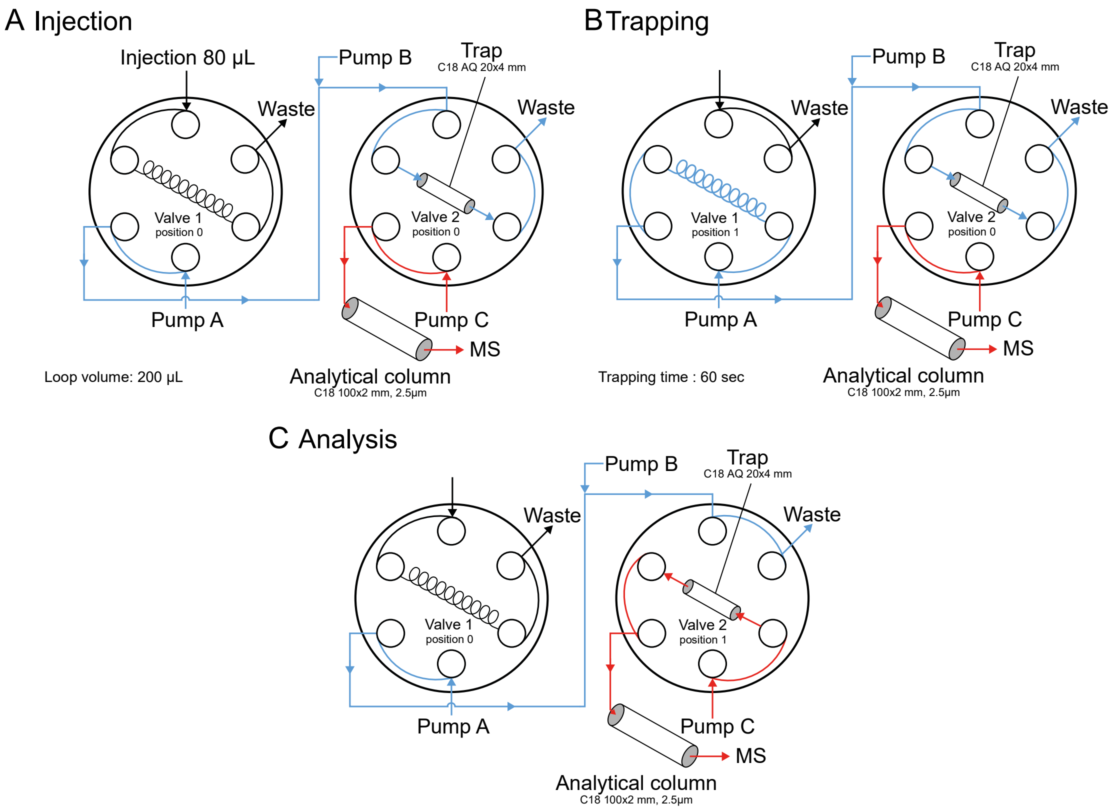

Figure S1. Schematic of column switching and online dilution scheme across three steps, injection (A), trapping (B), analysis (C). Valve 1 was a six-port valve and for valve 2 either a 6 or a 10-port valve were used.

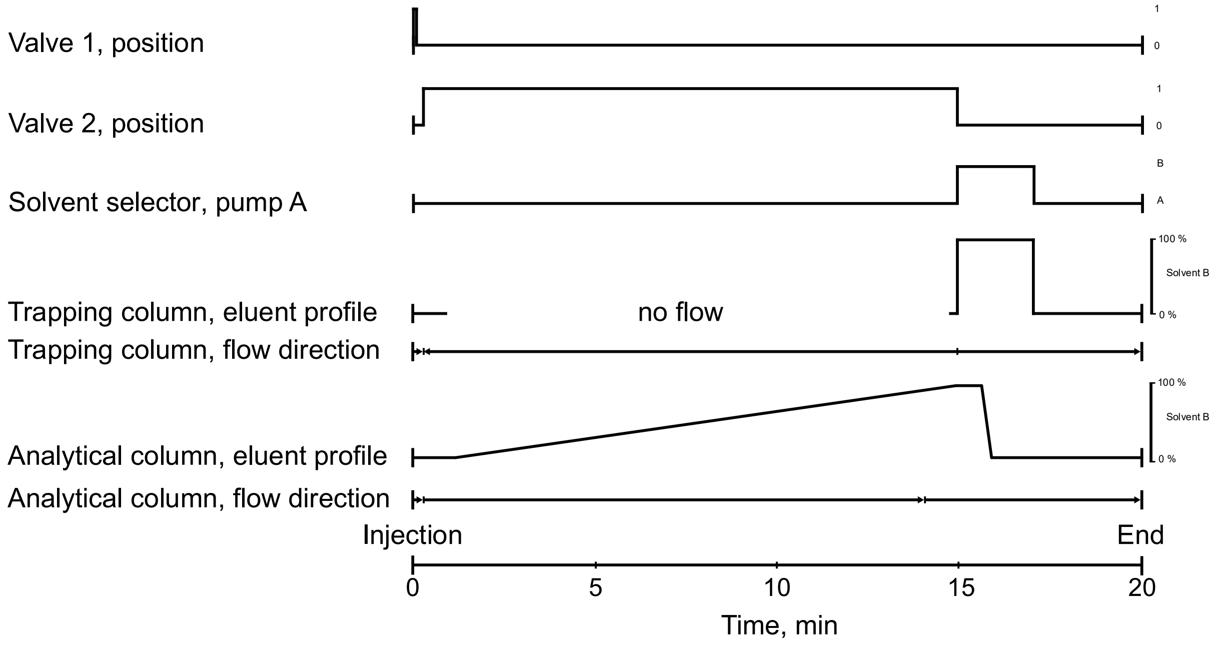

Figure S2. Column switching valves positions and timing along the acquisition.

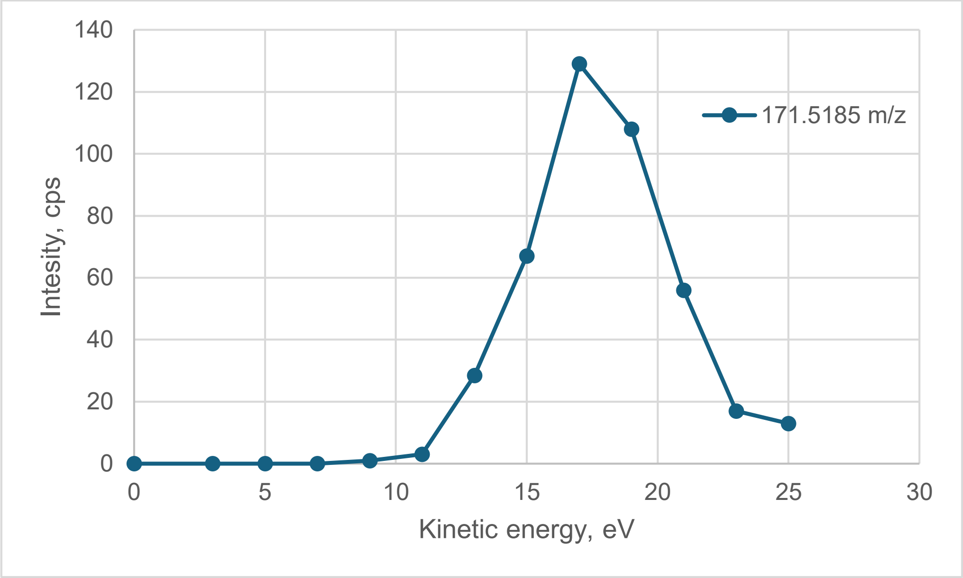

Figure S3. Boscalid product ion fragment (m/z 171.5181) intensity versus kinetic energy.

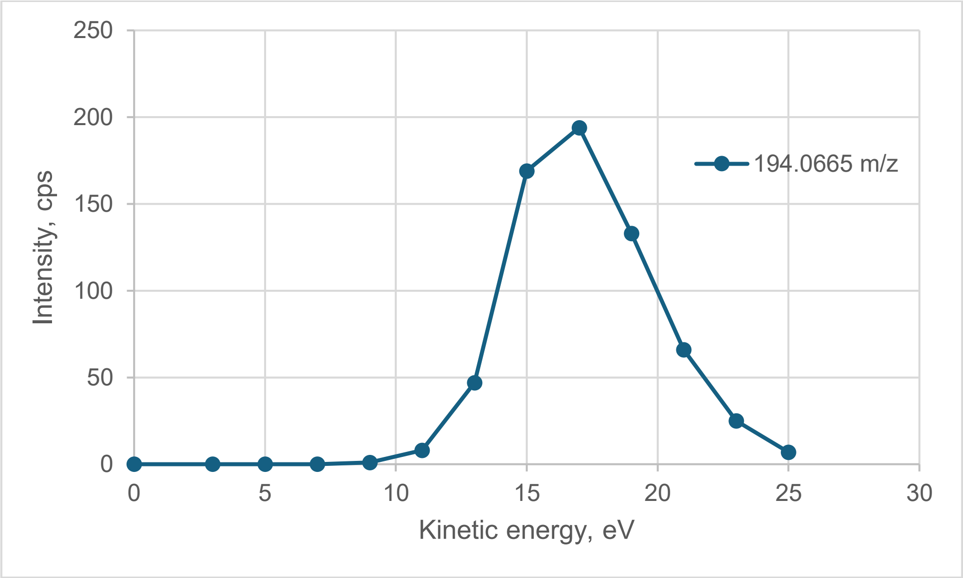

Figure S4. Dimethomorph product ion fragment (m/z 194.0655) intensity versus kinetic energy.

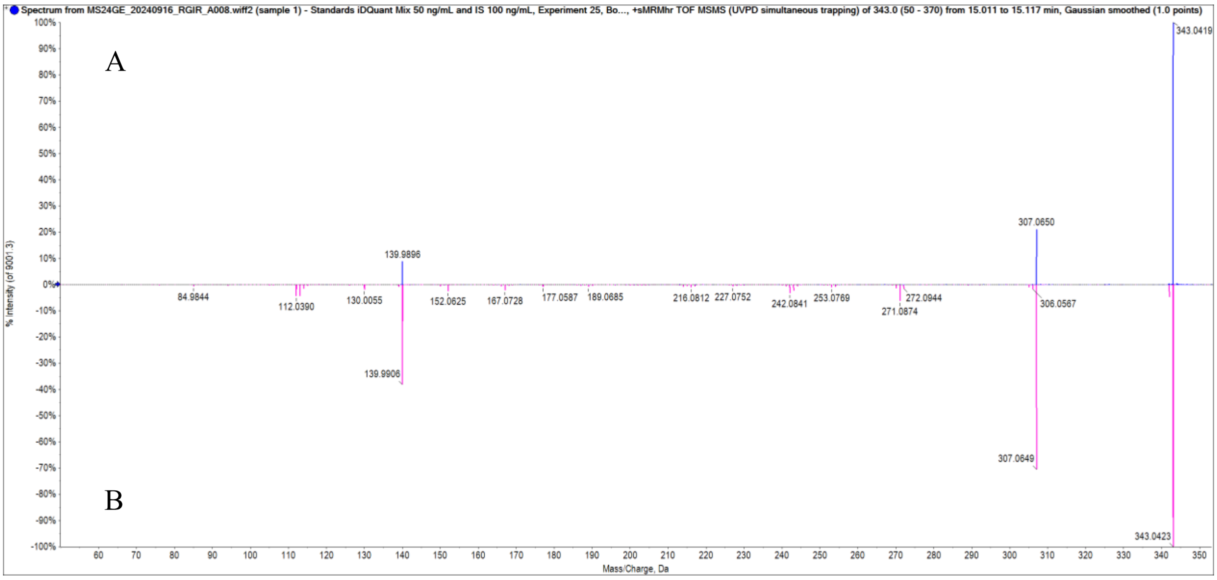

Figure S5. MRM HR UVPD spectra of boscalid at 266 nm (A) and 213 nm (B).

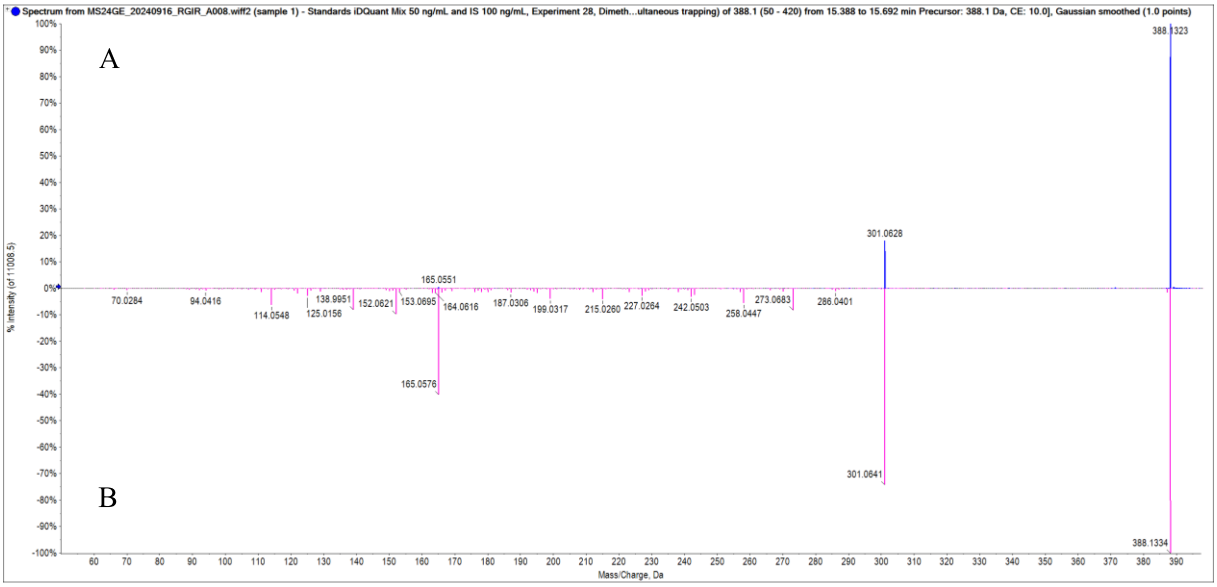

Figure S6. MRM HR UVPD spectrum of dimethomorph at 266 nm (A) and 213 nm (B).

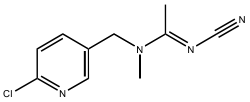

Acetamiprid
$C_{10}H_{11}ClN_4$
$[M+H]^+$= 223.0750 m/z

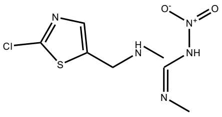

Clothianidin
$C_6H_8ClN_5O_2S$
$[M+H]^+$= 250.0165 m/z

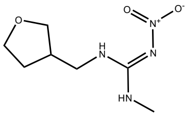

Dinotefuran
$C_7H_{14}N_4O_3$
$[M+H]^+$= 203.1144 m/z

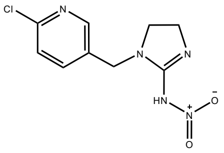

Imidacloprid
$C_9H_{10}ClN_5O_2$
$[M+H]^+$= 256.0601 m/z

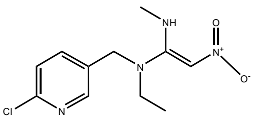

Nitenpyram
$C_{11}H_{15}ClN_4O_2$
$[M+H]^+$= 271.0961 m/z

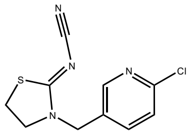

Thiacloprid
$C_{10}H_9ClN_4S$
$[M+H]^+$= 253.0314 m/z

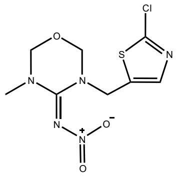

Thiamethoxam
$C_8H_{10}ClN_5O_3S$
$[M+H]^+$= 292.0271 m/z

Compound	pKa value at 25 ˚C[1]	LogP (pH 7, 20 C)
Acetamiprid	0.70	0.80
Imidacloprid	1.56	0.57
Nitenpyram	3.10	-0.66
Thiacloprid	-	1.26
Clothianidin	11.1	0.91
Thiamethoxam	-	-0.13
Dinotefuran	12.6	-0.55

Figure S7. Structures of the seven neonicotinoids investigated and their pKa and LogP values.

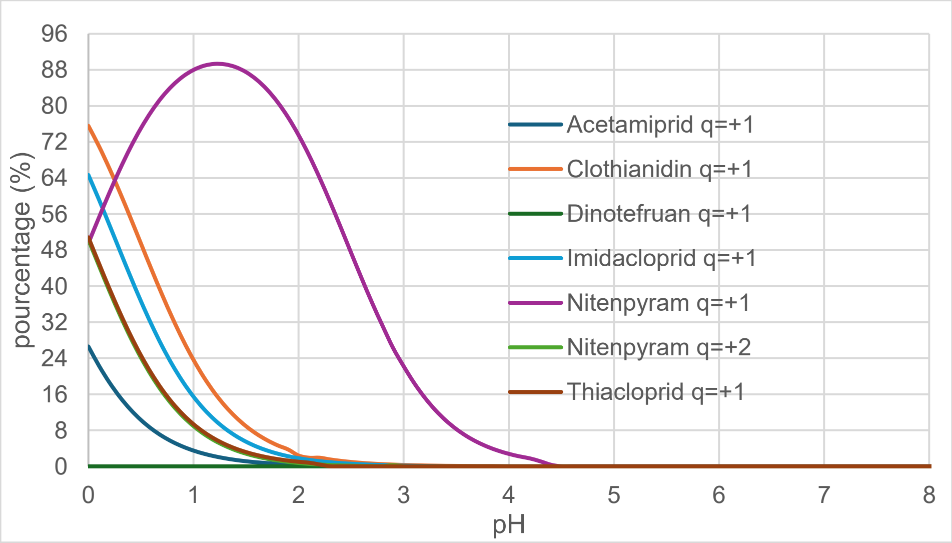

Figure S8. Predicted analyte charge states in function of pH (ACD lab Percepta 2012).

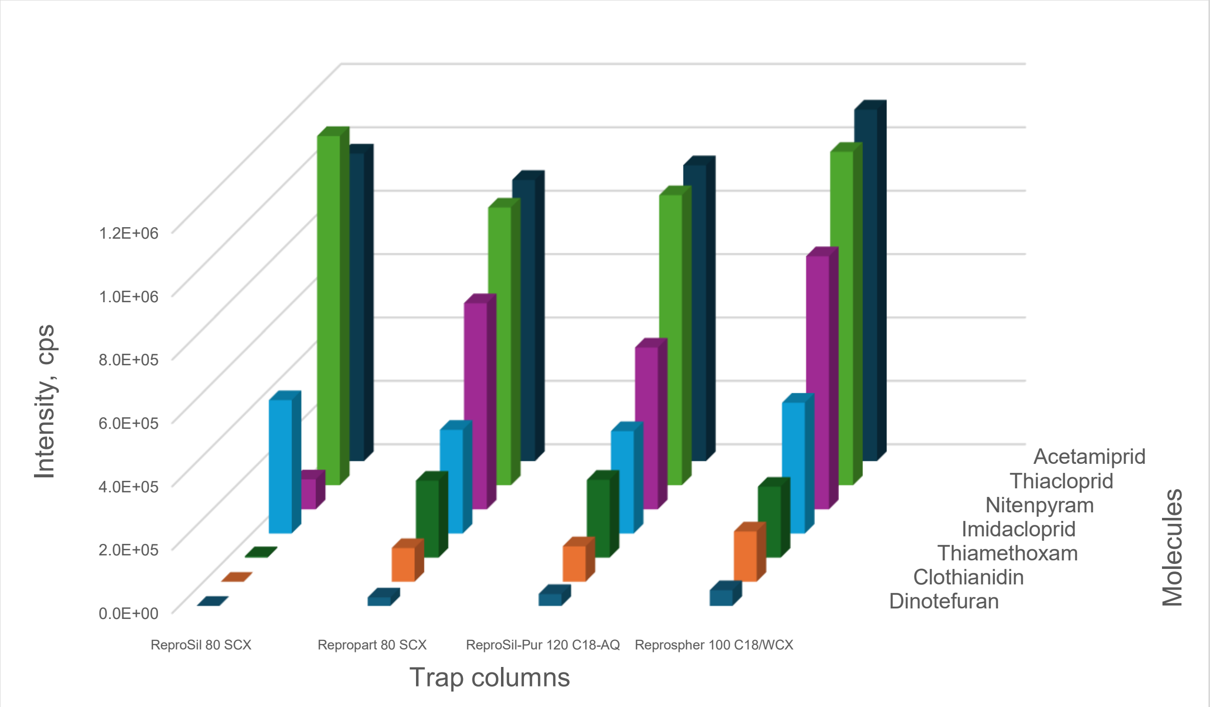

Figure S9. Signal intensity of seven neonicotinoids for different trap columns, trapping mobile phase 5 mM ammonium formate, injection volume of 40 µL. The analytes were dissolved in a mixture H2O/EtOH 85/15 (v/v) and 5 mM ammonium formate. Reprosil 80 SCX (RSil-80), Repropart 80 SCX (RP-80), Reprosil-Pur 120 C18-AQ (RSil-120), Reprospher 100 C18/WCX (RS-100).

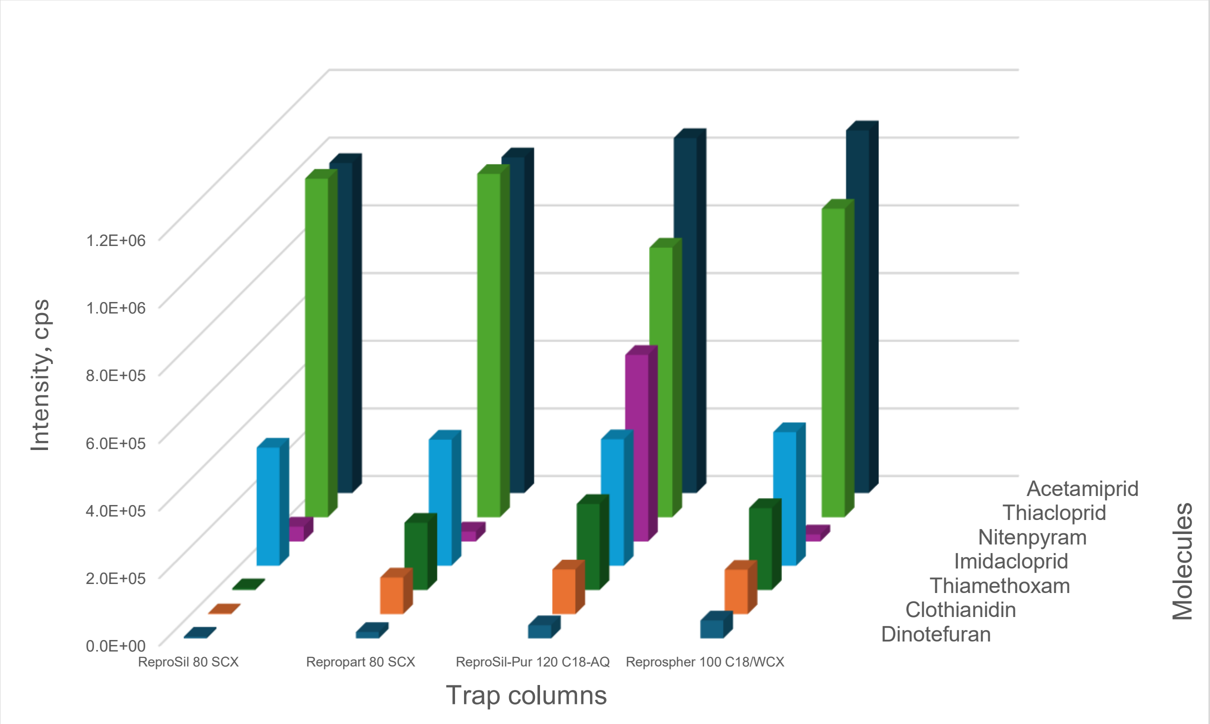

Figure S10. Signal intensity of seven neonicotinoids for different trap columns, trapping mobile phase 5 mM ammonium formate, injection volume of 40 µL. The analytes were dissolved in a mixture H2O/EtOH 85/15 (v/v) and 1% formic acid. Reprosil 80 SCX (RSil-80), Repropart 80 SCX (RP-80), Reprosil-Pur 120 C18-AQ (RSil-120), Reprospher 100 C18/WCX (RS-100).

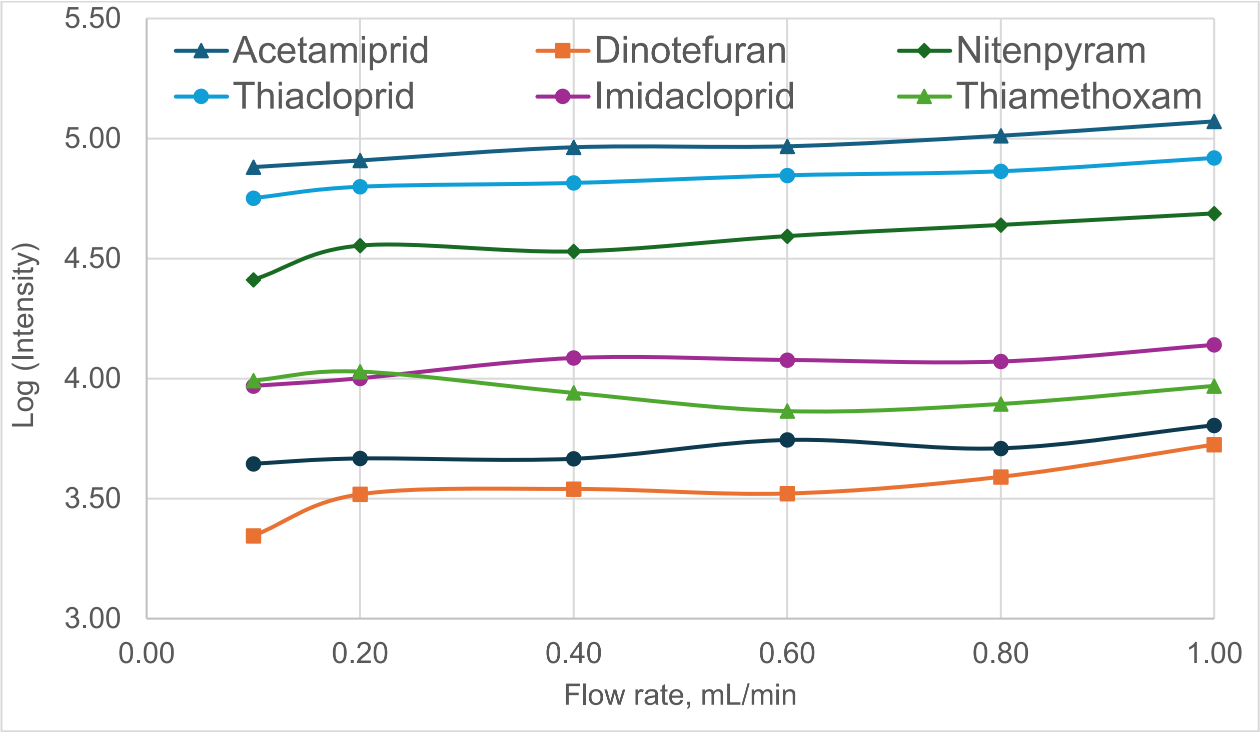

Figure S11. Pesticides signal intensities spiked in red wine in function on-line dilution flow rate. Injection flow rate was of 0.1 ml/min.

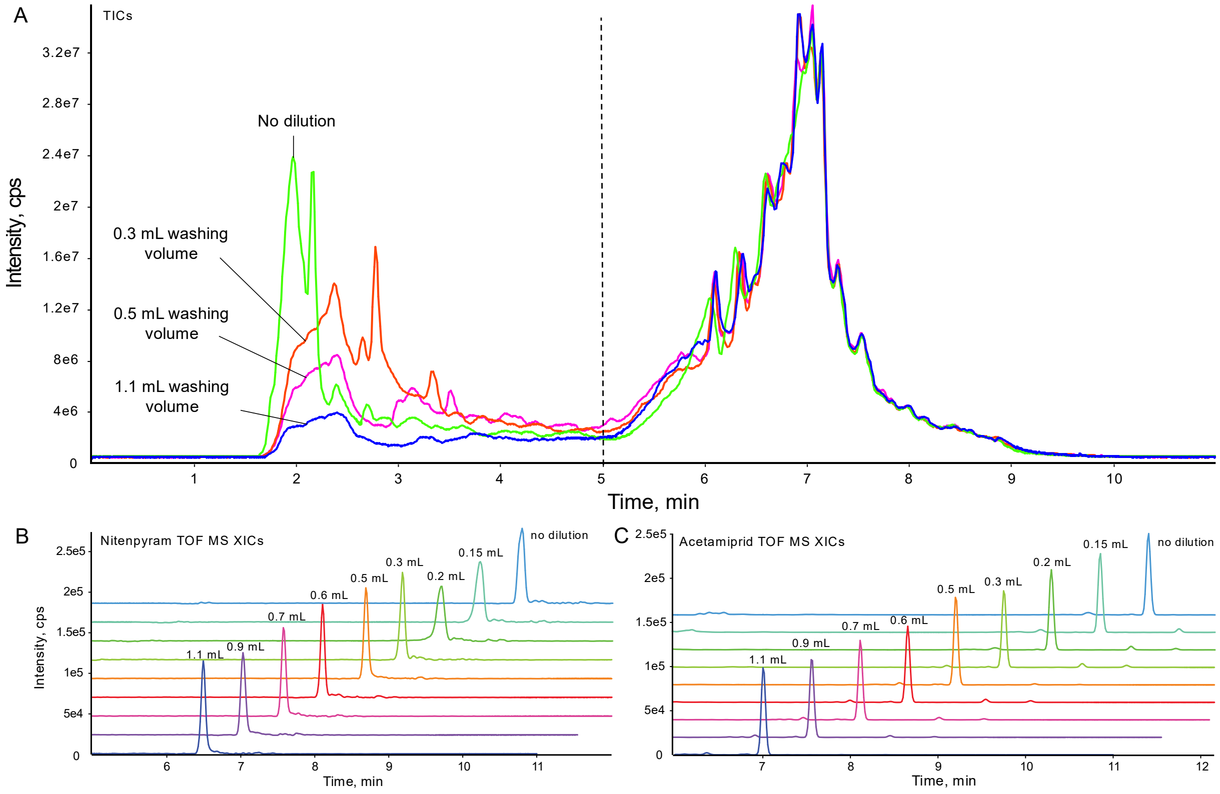

Figure S12. Impact of the washing volume on the TICs (A), and peak shape as observed in the XICs for the analytes nitenpyram, RT 6.4 min (B) and acetamiprid RT 7.0 min (C), injection volume 40 µL.

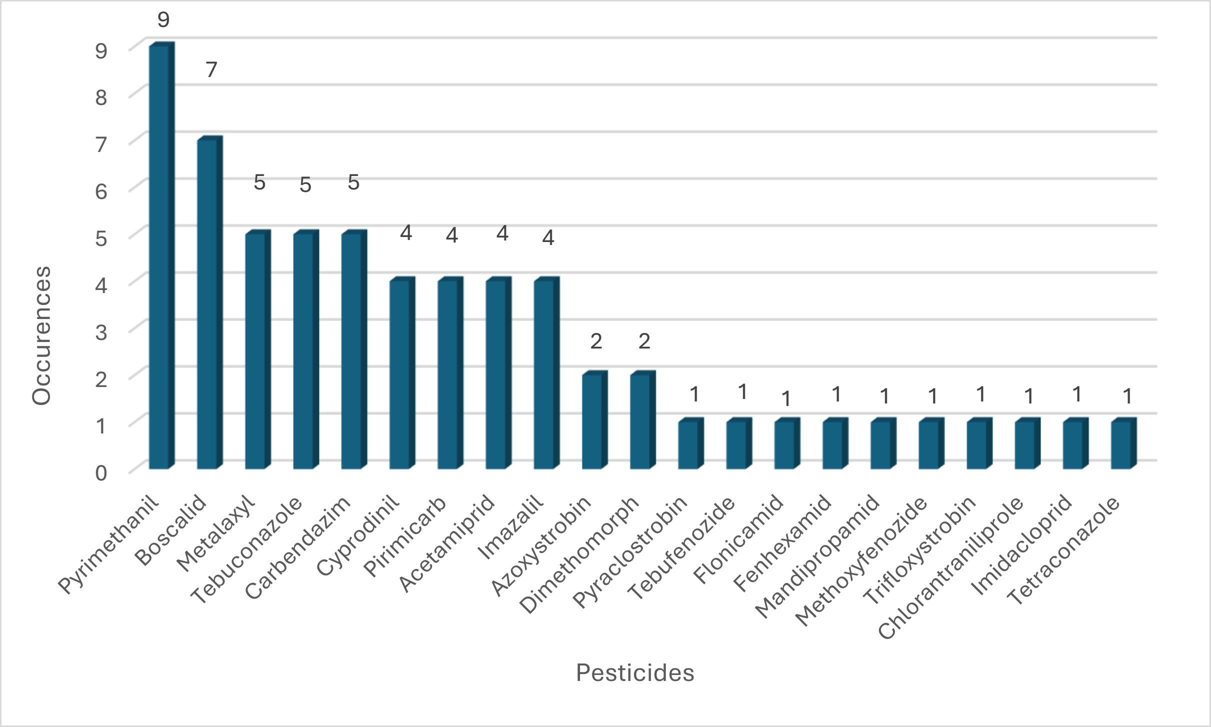

Figure S13. Frequency of pesticides found in juices.

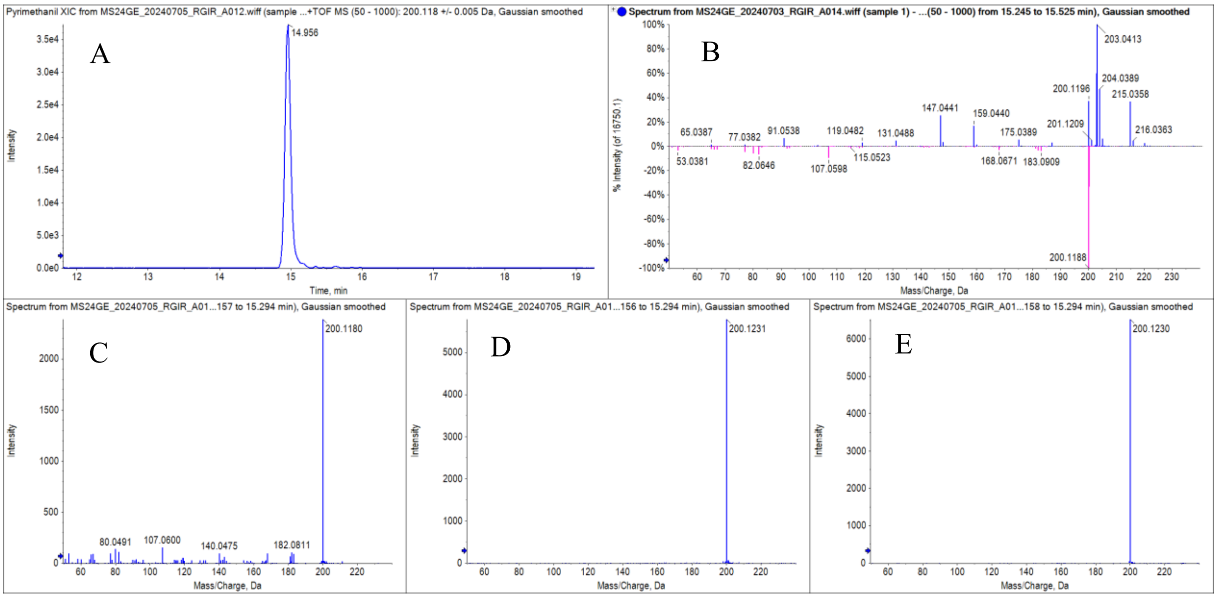

Figure S14. Detection of pyrimethanil in grapefruit juice sample J15 A) TOF MS XIC of *m/z* 280.154. B) Upper trace CID SWATH/MS spectrum of peak at RT = 14.9 and lower trace SWATH MS/MS CID spectrum of pyrimethanil C) MRMHR CID D), MRMHR EAD and E) MRMHR UVPD spectra of peak at RT = 14.9 in sample J16.

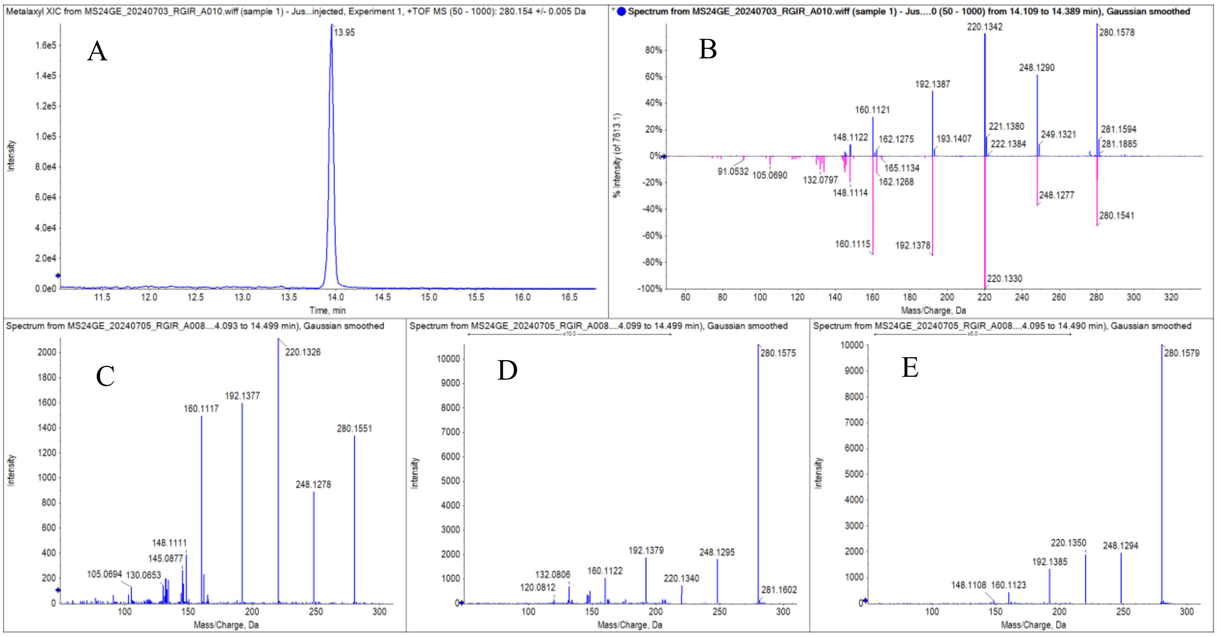

Figure S15. Detection of metalaxyl in grape juice sample J16 A) TOF MS XIC of m/z 280.154. B) Upper trace CID SWATH/MS spectrum of peak at RT = 13.9 and lower trace SWATH MS/MS CID spectrum of metalaxyl C) MRMHR CID D), MRMHR EAD and E) MRMHR UVPD spectra of peak at RT = 13.9 in sample J16.

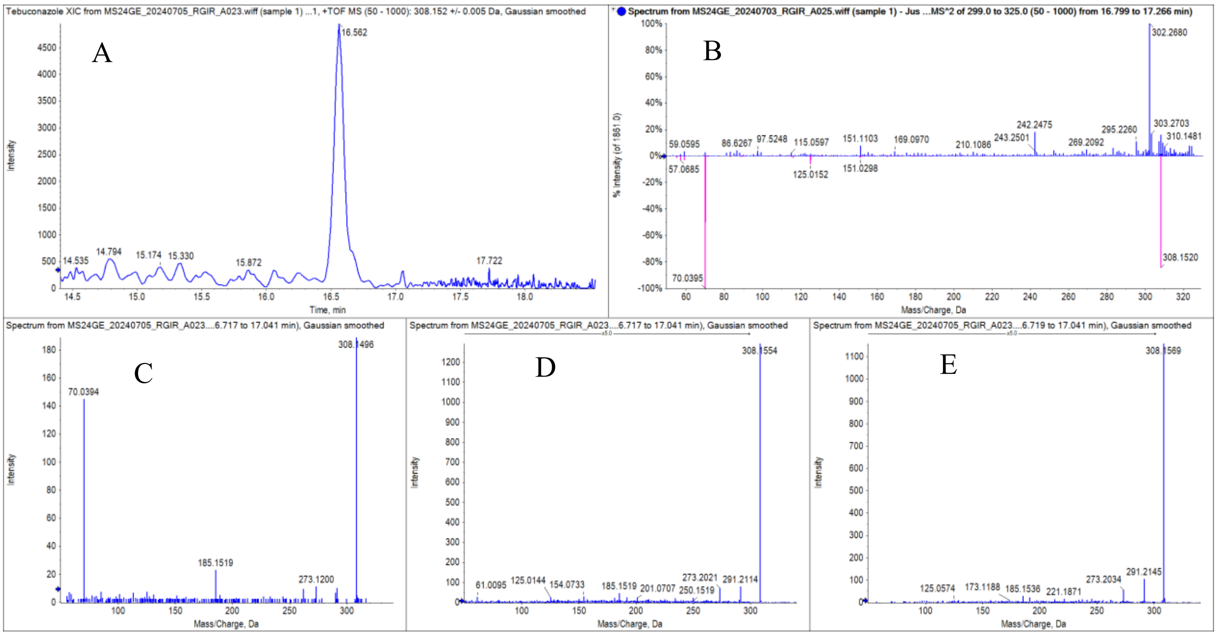

Figure S16. Detection of tebuconazole in multifruit juice sample J3 A) TOF MS XIC of *m/z* 308.152. B) Upper trace CID SWATH/MS spectrum of peak at RT = 16.8 and lower trace SWATH MS/MS CID spectrum of tebuconazole C) MRMHR CID D), MRMHR EAD and E) MRMHR UVPD spectra of peak at RT = 16.8 in sample J3.

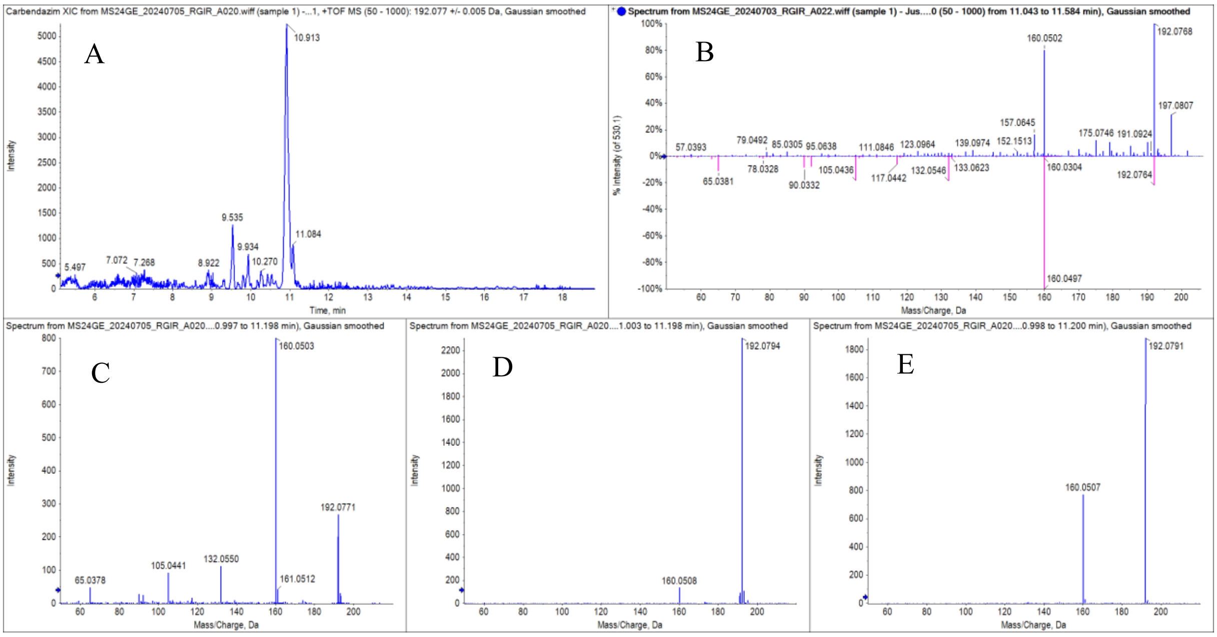

Figure S17. Detection of carbendazim in apple juice J9 A) TOF MS XIC of *m/z* 192.077. B) Upper trace CID SWATH/MS spectrum of peak at RT = 10.9 and lower trace SWATH MS/MS CID spectrum of carbendazim C) MRMHR CID D), MRMHR EAD and E) MRMHR UVPD spectra of peak at RT = 10.9 in sample J9.

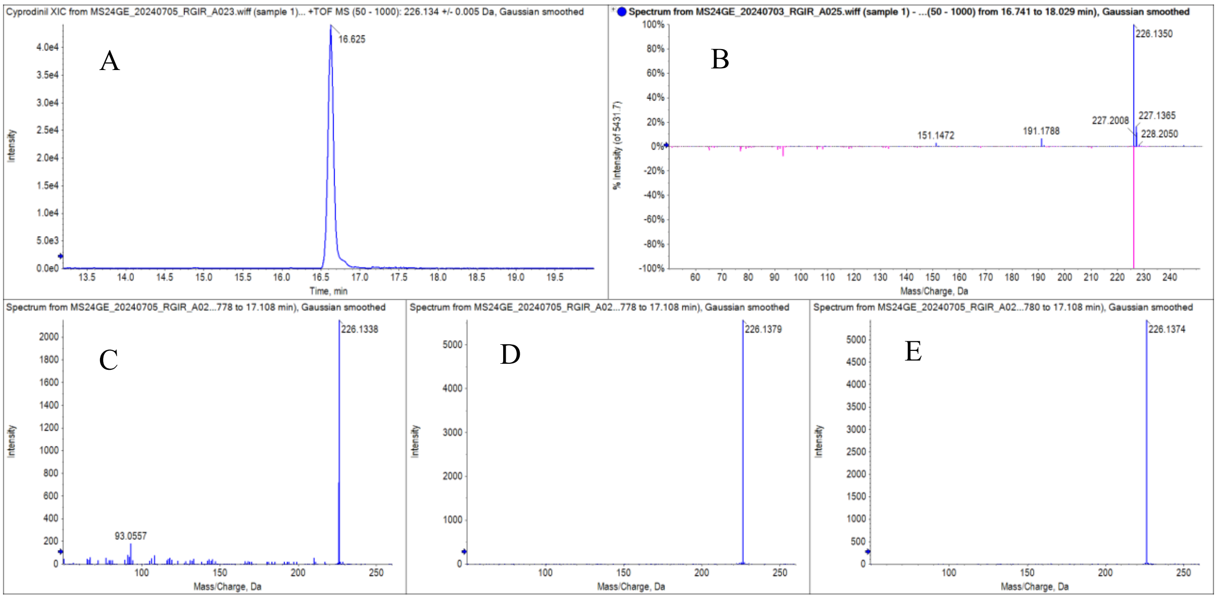

Figure S18. Detection of cyprodinil in multifruit juice sample J3 A) TOF MS XIC of *m/z* 226.134. B) Upper trace CID SWATH/MS spectrum of peak at RT = 16.6 and lower trace SWATH MS/MS CID spectrum of cyprodinil C) MRMHR CID D), MRMHR EAD and E) MRMHR UVPD spectra of peak at RT = 16.6 in sample J3.

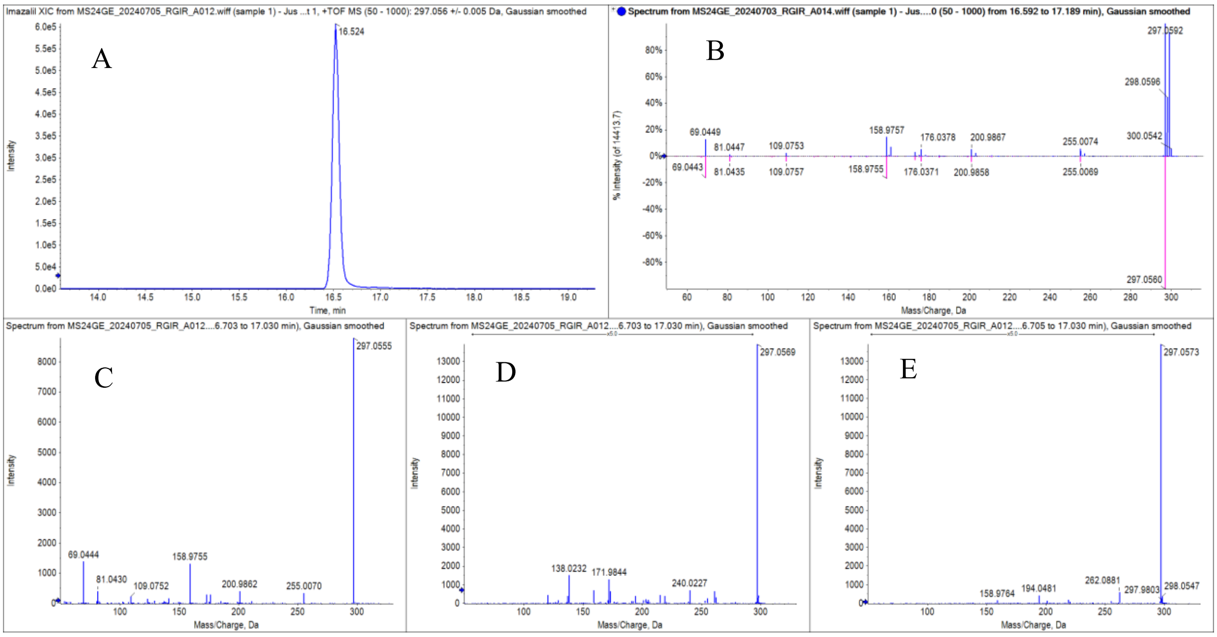

Figure S19. Detection of imazalil in grapefruit juice sample J15 A) TOF MS XIC of *m/z* 297.056. B) Upper trace CID SWATH/MS spectrum of peak at RT = 16.5 and lower trace SWATH MS/MS CID spectrum of imazalil C) MRMHR CID D), MRMHR and E) MRMHR UVPD spectra of peak at RT = 16.5 in sample J15.

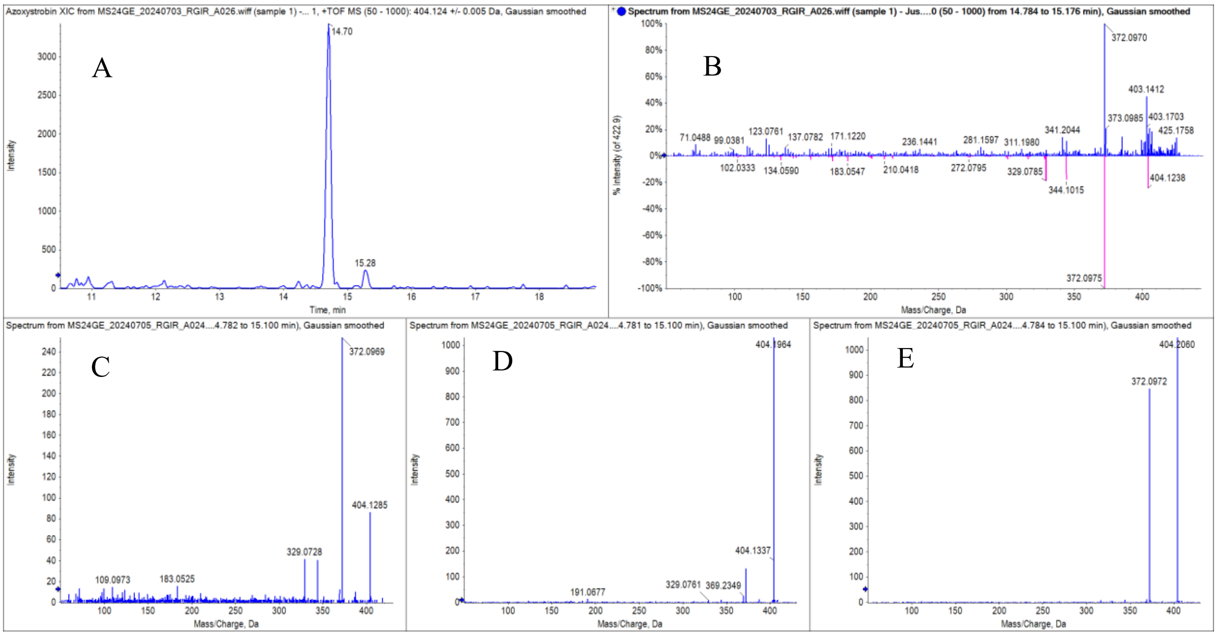

Figure S20. Detection of azoxystrobin in pomegranate juice sample J7 A) TOF MS XIC of *m/z* 404.124. B) Upper trace CID SWATH/MS spectrum of peak at RT = 14.7 and lower trace SWATH MS/MS CID spectrum of azoxystrobin C) MRMHR CID D), MRMHR and E) MRMHR UVPD spectra of peak at RT = 14.7 in sample J7.

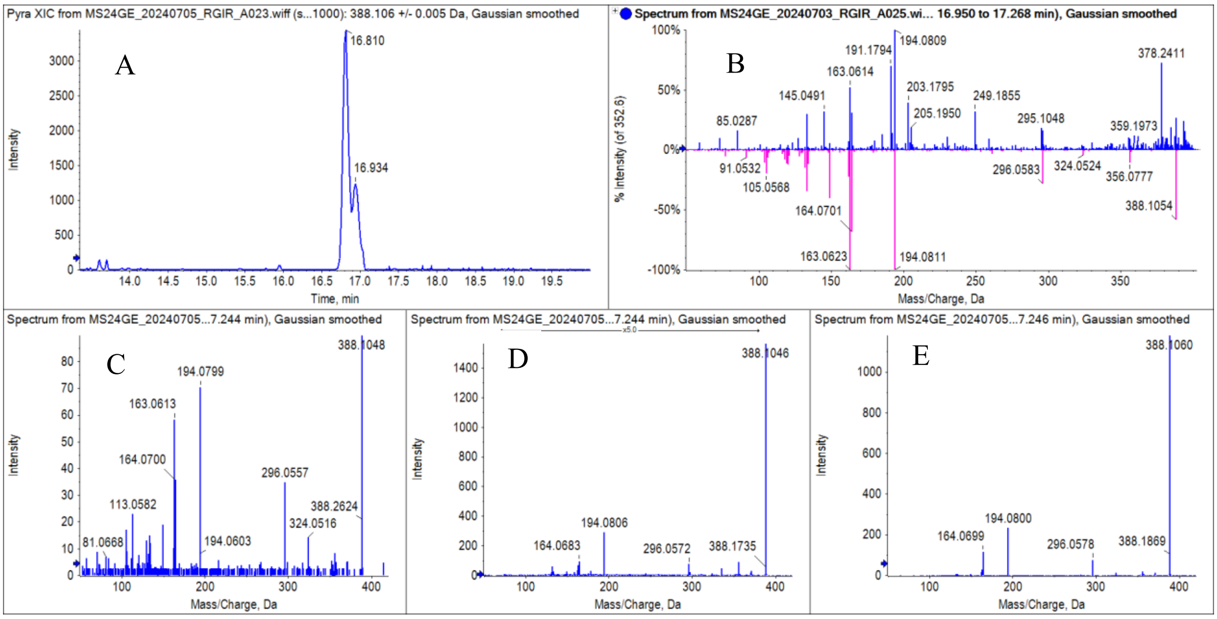

Figure S21. Detection of pyraclostrobin in multifruit juice sample J3 A) TOF MS XIC of *m/z* 388.106. B) Upper trace CID SWATH/MS spectrum of peak at RT = 16.8 and lower trace SWATH MS/MS CID spectrum of pyraclostrobin C) MRMHR CID D), MRMHR EAD and E) MRMHR UVPD spectra of peak at RT = 16.8 in sample J3.

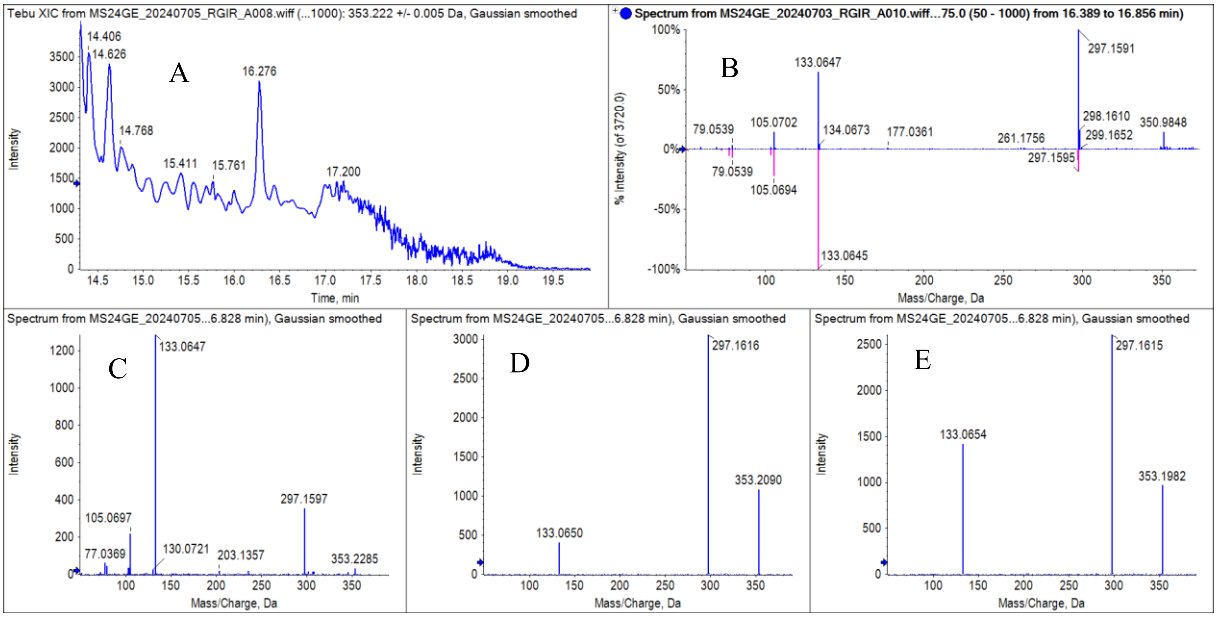

Figure S22. Detection tebufenozide in grape juice sample J16 A) TOF MS XIC of *m/z* 353.22. B) Upper trace CID SWATH/MS spectrum of peak at RT = 16.3 and lower trace SWATH MS/MS CID spectrum of tebufenozide C) MRMHR CID D), MRMHR EAD and E) MRMHR UVPD spectra of peak at RT = 16.3 in sample J16.

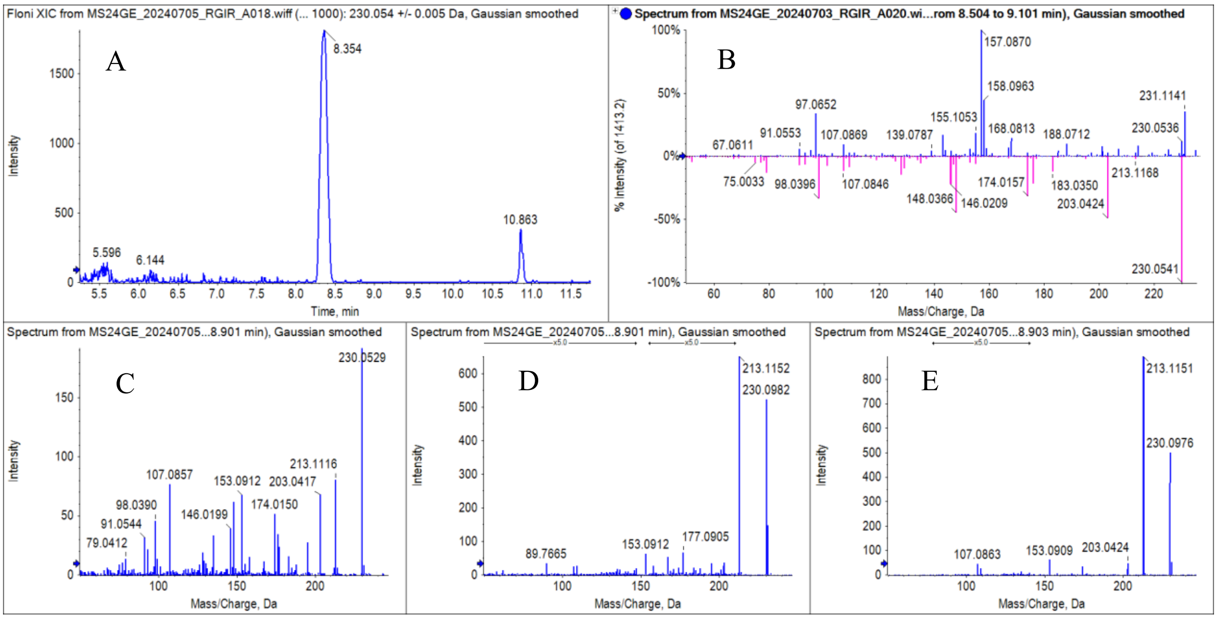

Figure S23. Detection of flonicamid in apple juice sample J8 A) TOF MS XIC of *m/z* 230.054. B) Upper trace CID SWATH/MS spectrum of peak at RT = 8.3 and lower trace SWATH MS/MS CID spectrum of flonicamid C) MRMHR CID D), MRMHR EAD and E) MRMHR UVPD spectra of peak at RT = 8.3 in sample J8.

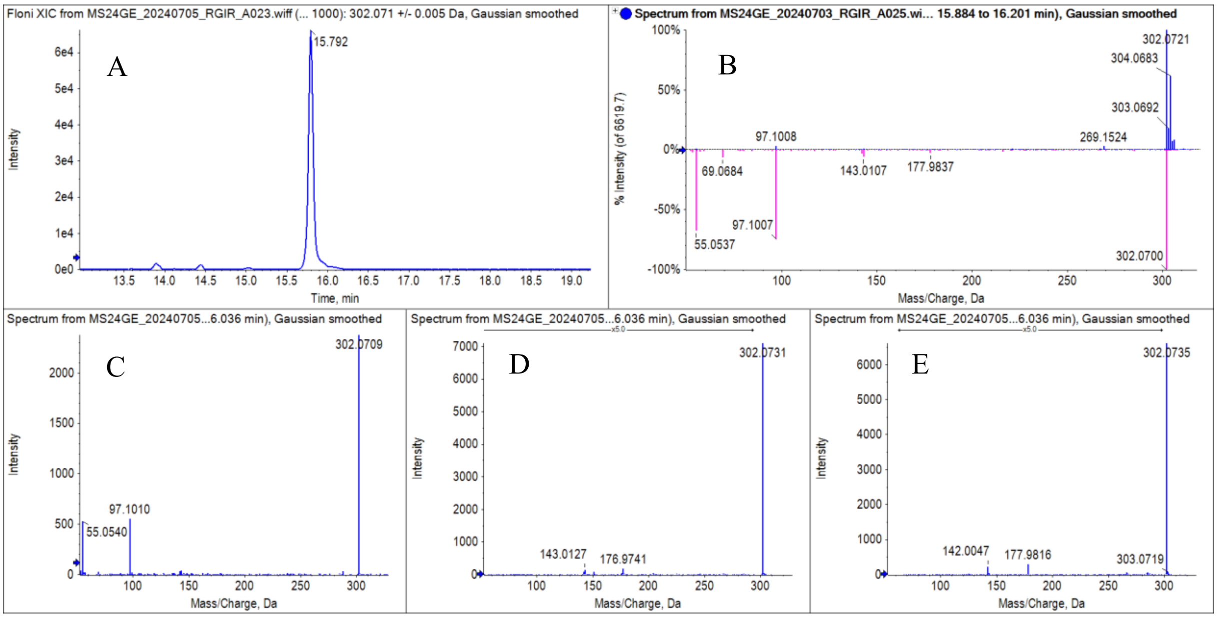

Figure S24. Detection of fenhexamid in tomato juice sample J17 A) TOF MS XIC of *m/z* 302.071. B) Upper trace CID SWATH/MS spectrum of peak at RT = 15.8 and lower trace SWATH MS/MS CID spectrum of fenhexamid C) MRMHR CID D), MRMHR EAD and E) MRMHR UVPD spectra of peak at RT = 15.8 in sample J17.

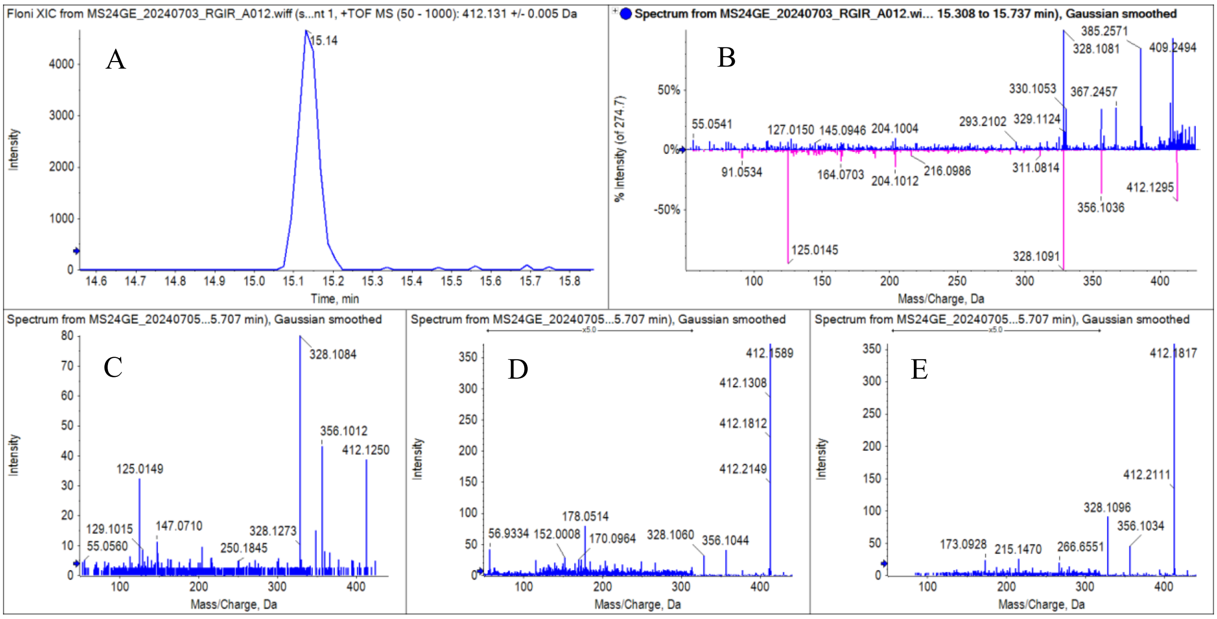

Figure S25. Detection of mandipropamid in tomato juice sample J17 A) TOF MS XIC of *m/z* 412.131. B) Upper trace CID SWATH/MS spectrum of peak at RT = 15.1 and lower trace SWATH MS/MS CID spectrum of mandipropamid C) MRMHR CID D), MRMHR EAD, and E) MRMHR UVPD spectra of peak at RT = 15.1 in sample J17.

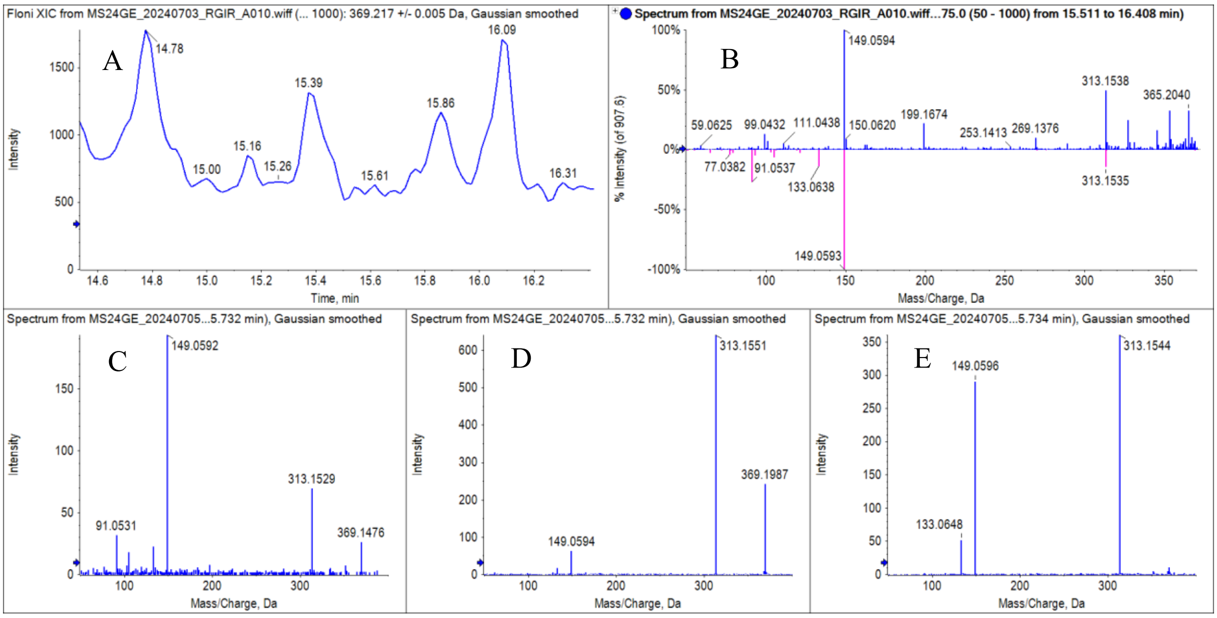

Figure S26. Detection of methoxyfenozide at in grape juice sample J16 A) TOF MS XIC of *m/z* 369.217 B) Upper trace CID SWATH/MS spectrum of peak at RT = 15.9 and lower trace SWATH MS/MS CID spectrum of methoxyfenozide C) MRMHR spectra CID D), MRMHR EAD, and E) MRMHR UVPD spectra of peak at RT = 17.2 in sample J16.

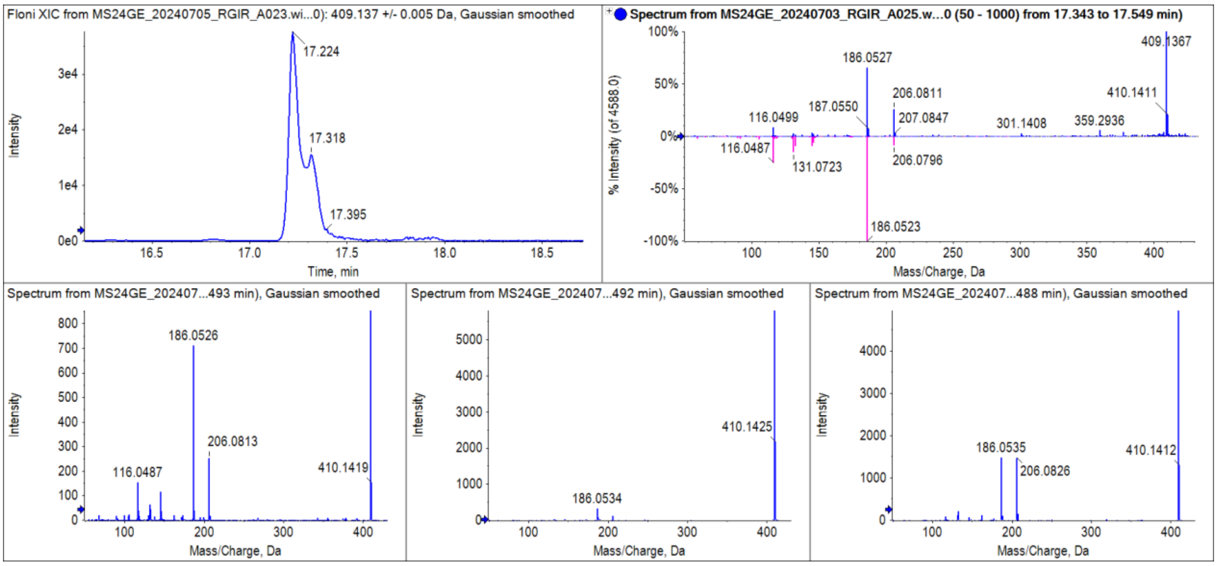

Figure S27. Detection of trifloxystrobin in multifruit juice sample J3 A) TOF MS XIC of *m/z* 409.137 B) Upper trace CID SWATH/MS spectrum of peak at RT = 17.2 and lower trace SWATH MS/MS CID spectrum of trifloxystrobin C) MRMHR CID D), MRMHR EAD, and E) MRMHR UVPD spectra of peak at RT = 17.2 in sample J3.

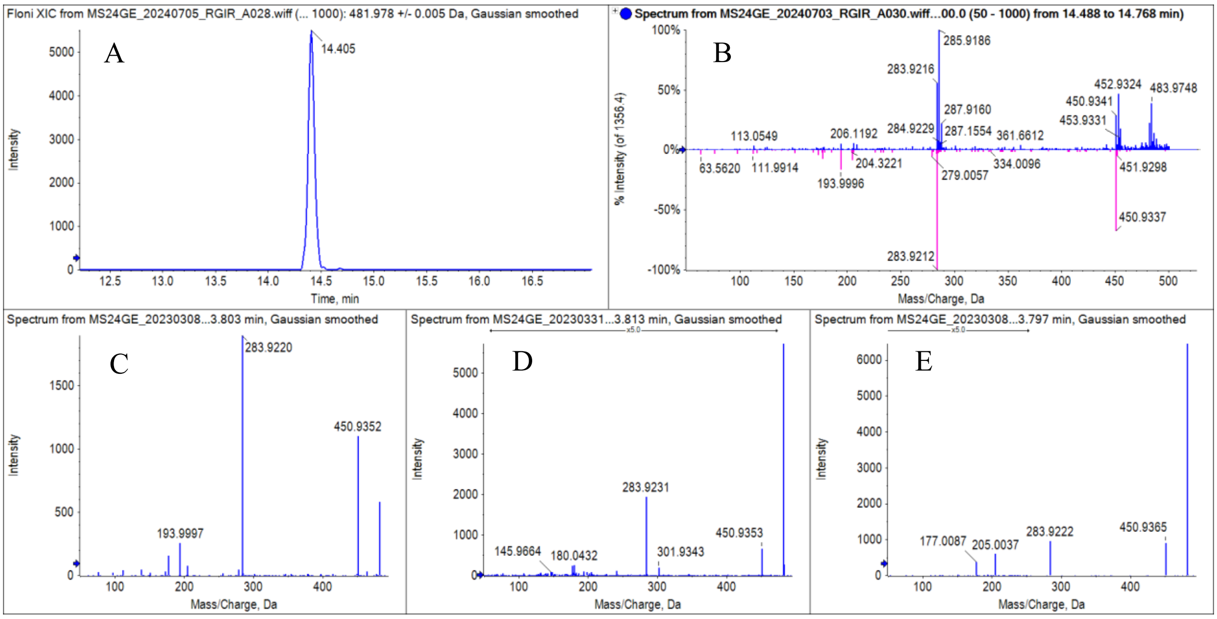

Figure S28. Detection of chlorantraniliprole in apple juice sample J20 A) TOF MS XIC of *m/z* 481.978 B) Upper trace CID SWATH/MS spectrum of peak at RT = 14.4 and lower trace SWATH MS/MS CID spectrum of thlorantraniliprole C) MRMHR CID D), MRMHR EAD, and E) MRMHR UVPD spectra of peak at RT = 14.4 in sample J20.

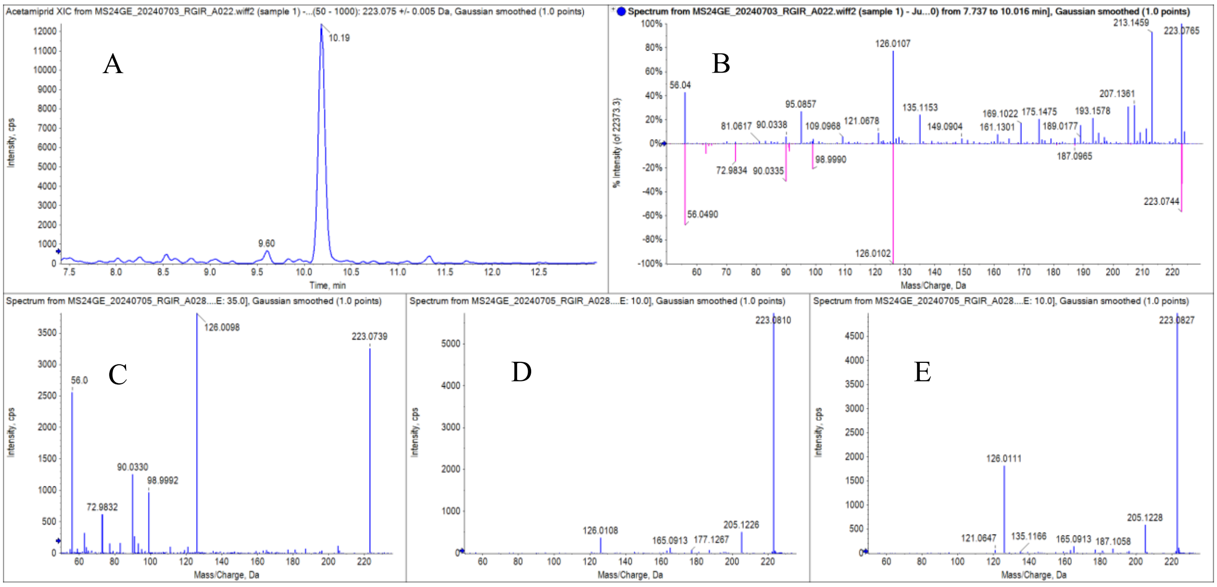

Figure S29. Detection of acetamiprid in apple juice sample J9 A) TOF MS XIC of *m/z* 223.075 B) Upper trace CID SWATH/MS spectrum of peak at RT = 10.2 and lower trace SWATH MS/MS CID spectrum of acetamiprid C) MRMHR CID D), MRMHR EAD, and E) MRMHR UVPD spectra of peak at RT = 10.2 in sample J9.

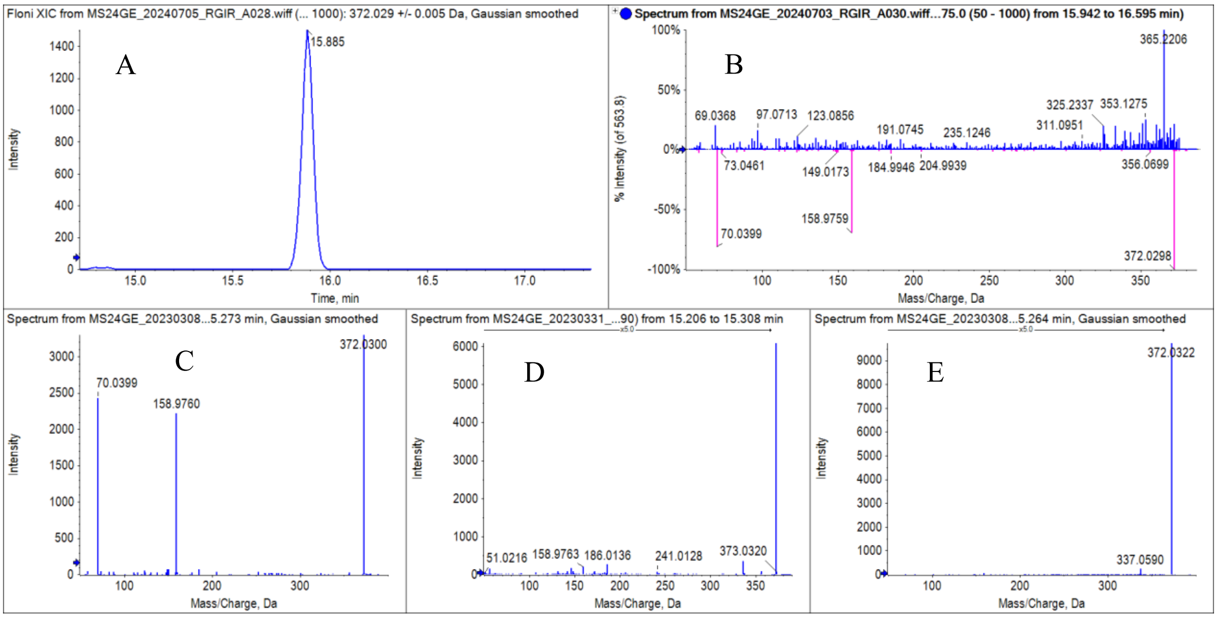

Figure S30. Detection of tetraconazole in apple juice sample J20 A) TOF MS XIC of *m/z* 372.029 B) Upper trace CID SWATH/MS spectrum of peak at RT = 15.9 and lower trace SWATH MS/MS CID spectrum of tetraconazole C) MRMHR CID D), MRMHR EAD, and E) MRMHR UVPD spectra of peak at RT = 15.9 in sample J20.

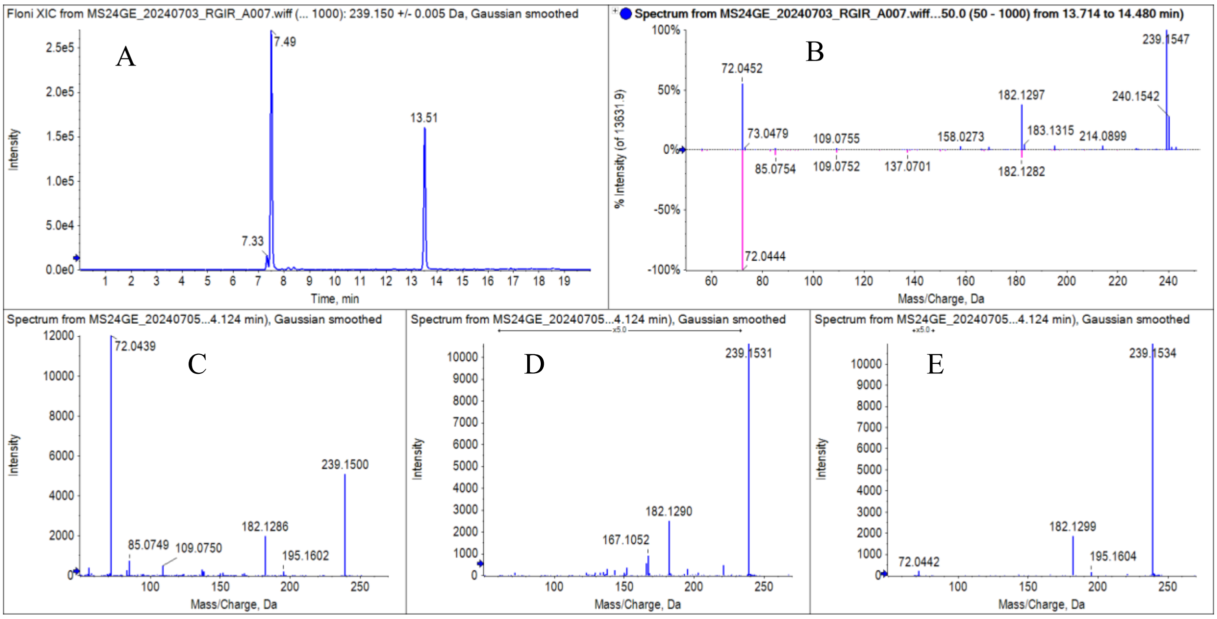

Figure S31. Detection of pirimicarb in apple juice sample J2 A) TOF MS XIC of *m/z* 239.150 B) Upper trace CID SWATH/MS spectrum of peak at RT = 13.5 and lower trace SWATH MS/MS CID spectrum of pirimicarb C) MRMHR CID D), MRMHR EAD, and E) MRMHR UVPD spectra of peak at RT = 13.5 in sample J2.

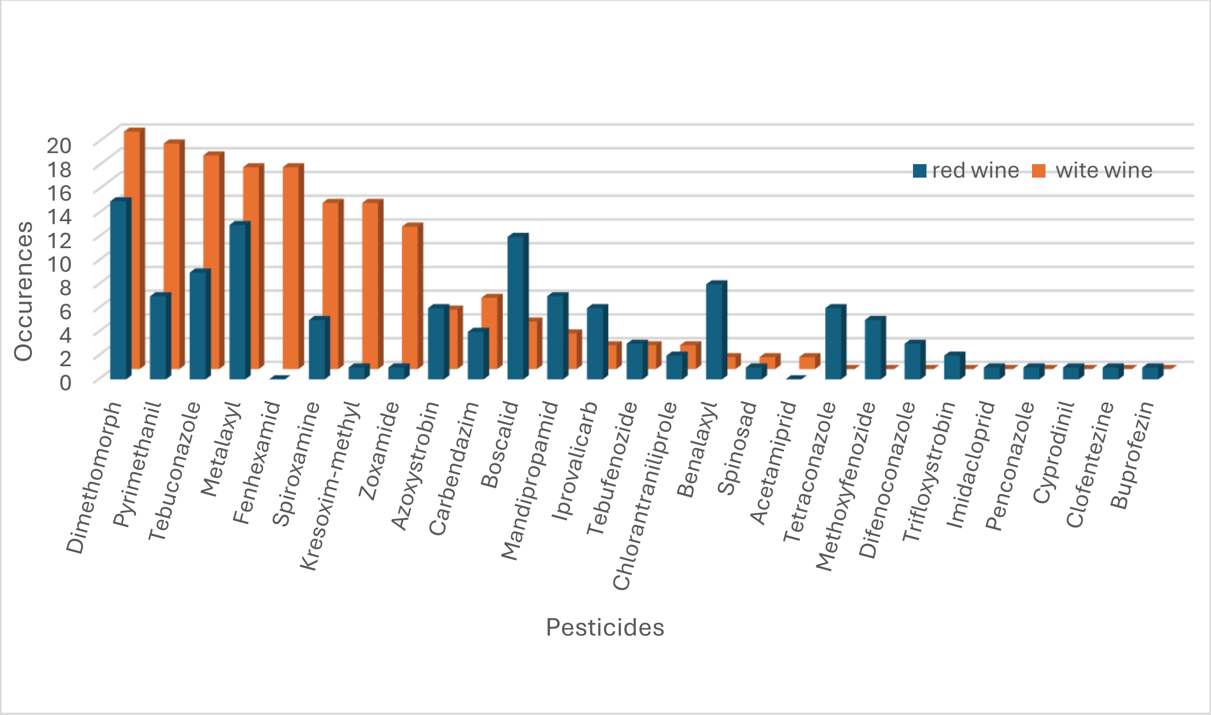

Figure S32. Frequency of pesticides found in red and white wines.

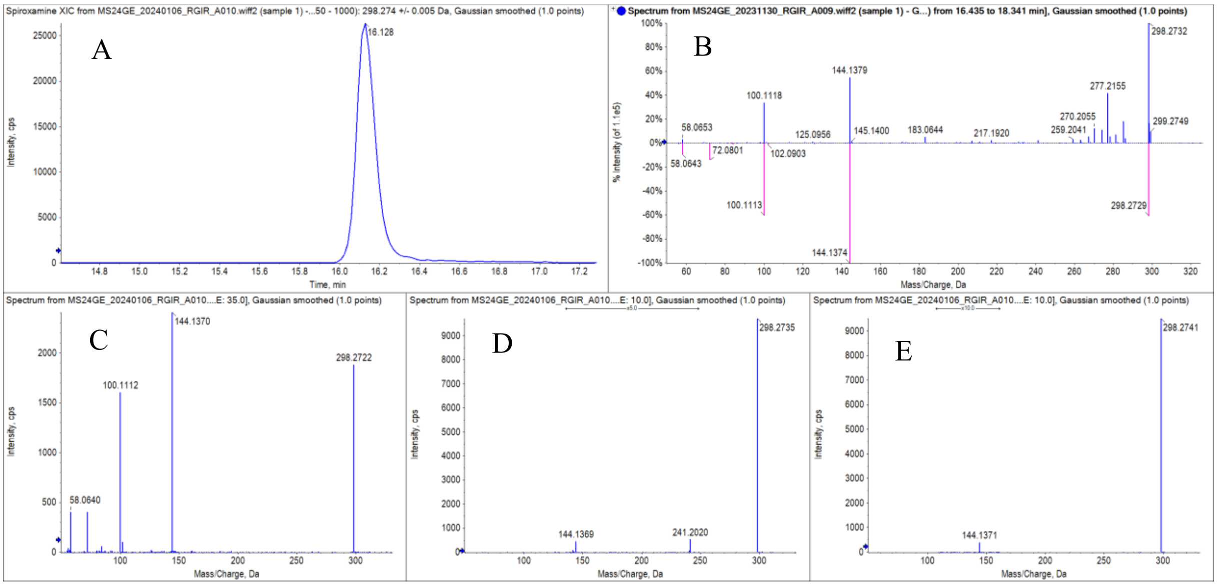

Figure S33. Detection of spiroxamine in a white wine sample W20 A) TOF MS XIC of *m/z* 239.150 B) Upper trace CID SWATH/MS spectrum of peak at RT = 16.1 and lower trace SWATH MS/MS CID spectrum of spiroxamine C) MRMHR CID D), MRMHR EAD, and E) MRMHR UVPD spectra of peak at RT = 16.1 in sample W20.

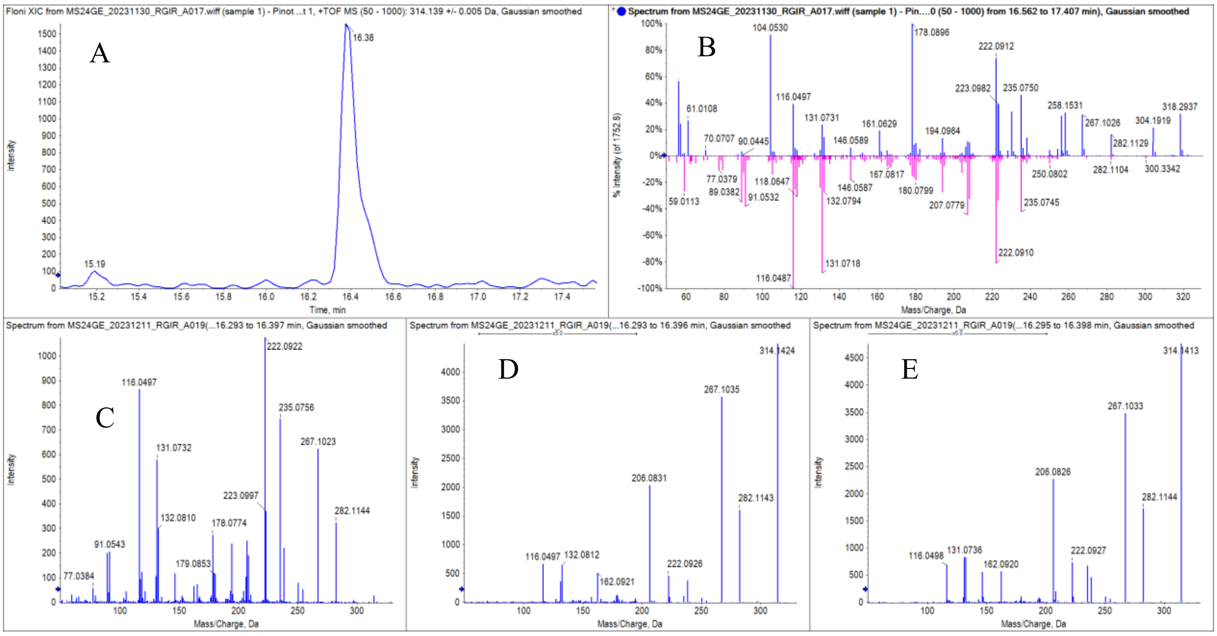

Figure S34. Detection of kresoxim-methyl in a white wine sample W31 A) TOF MS XIC of *m/z* 314.139 B) Upper trace CID SWATH/MS spectrum of peak at RT = 16.4 and lower trace SWATH MS/MS CID spectrum of spiroxamine C) MRMHR CID D), MRMHR EAD, and E) MRMHR UVPD spectra of peak at RT = 16.4 in sample W31.

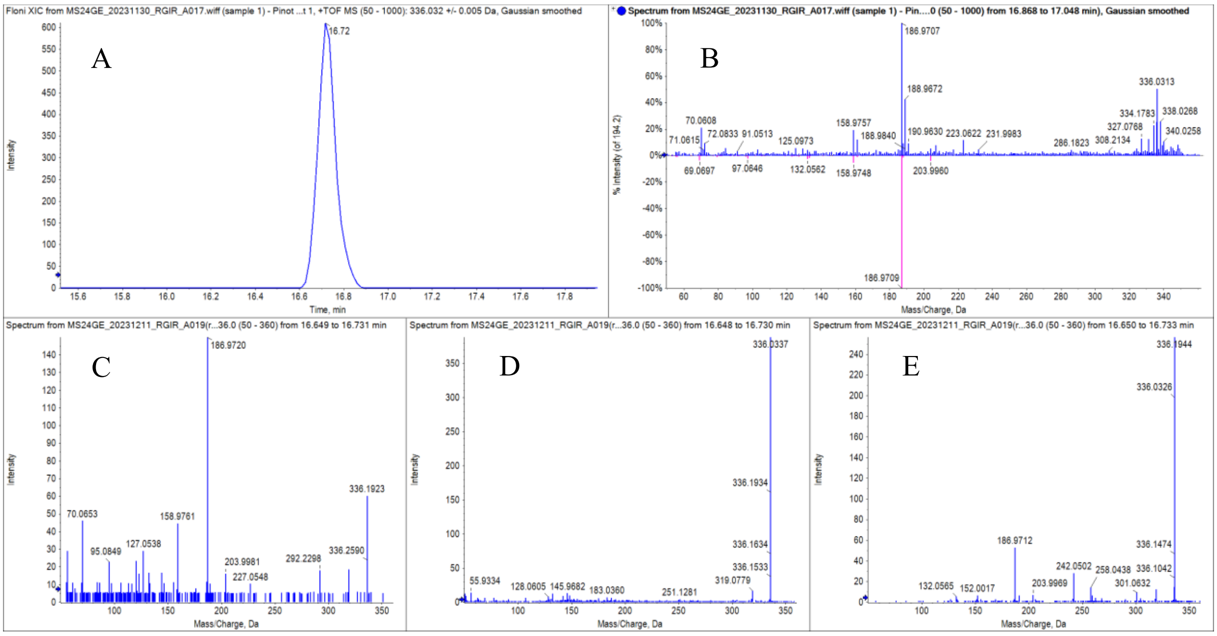

Figure S35. Detection of zoxamide in a white wine sample W31 A) TOF MS XIC of *m/z* 336.032 B) Upper trace CID SWATH/MS spectrum of peak at RT = 16.7 and lower trace SWATH MS/MS CID spectrum of zoxamide C) MRMHR CID D), MRMHR EAD, and E) MRMHR UVPD spectra of peak at RT = 16.7 in sample W31.

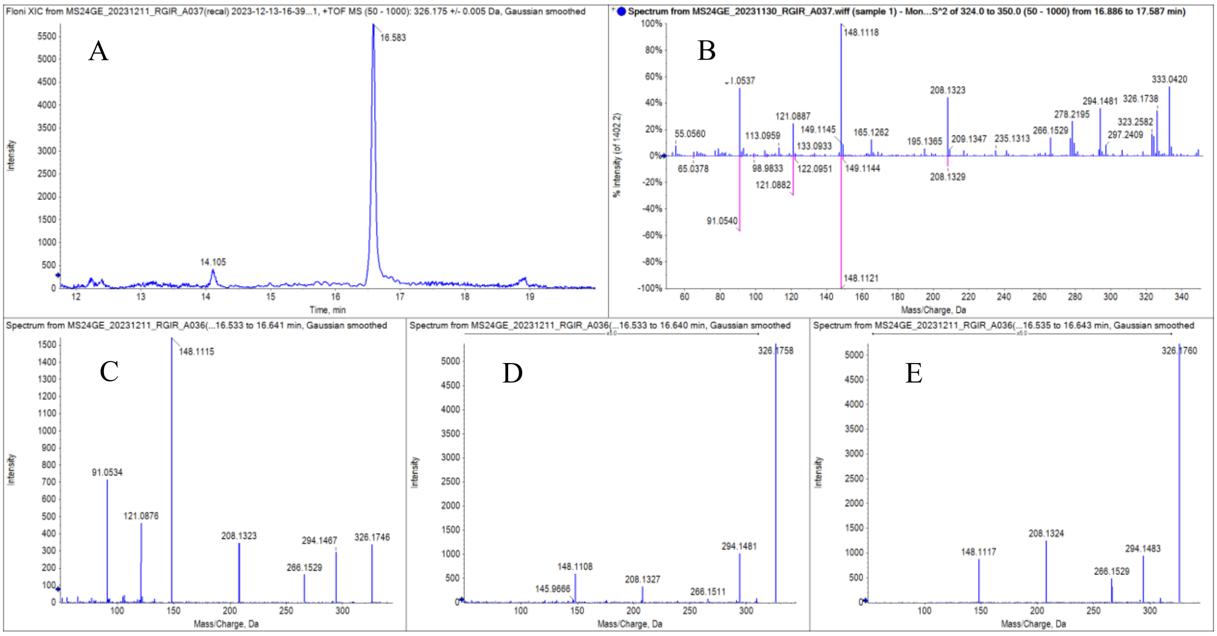

Figure S36. Detection of benalaxyl in a red wine sample W4 A) TOF MS XIC of *m/z* 326.175 B) Upper trace CID SWATH/MS spectrum of peak at RT = 16.6 and lower trace SWATH MS/MS CID spectrum of benalaxyl C) MRMHR CID D), MRMHR EAD, and E) MRMHR UVPD spectra of peak at RT = 16.6 in sample W4.

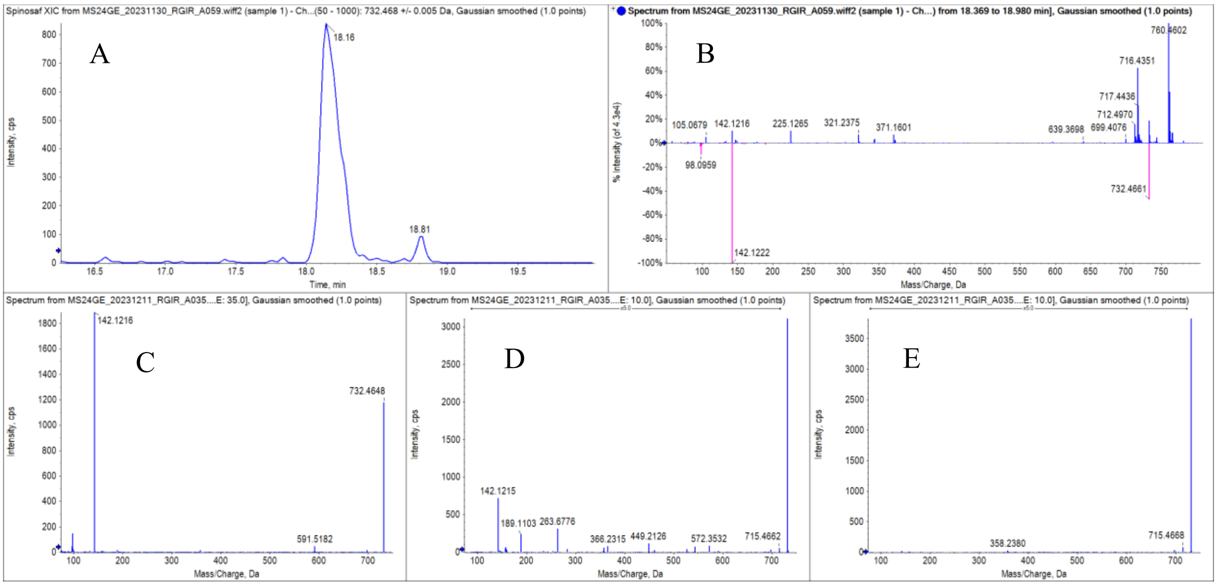

Figure S37. Detection of spinosad in a red wine sample W5 A) TOF MS XIC of *m/z* 732.468 B) Upper trace CID SWATH/MS spectrum of peak at RT = 18.2 and lower trace SWATH MS/MS CID spectrum of spinosad C) MRMHR CID D), MRMHR EAD, and E) MRMHR UVPD spectra of peak at RT = 18.2 in sample W5.

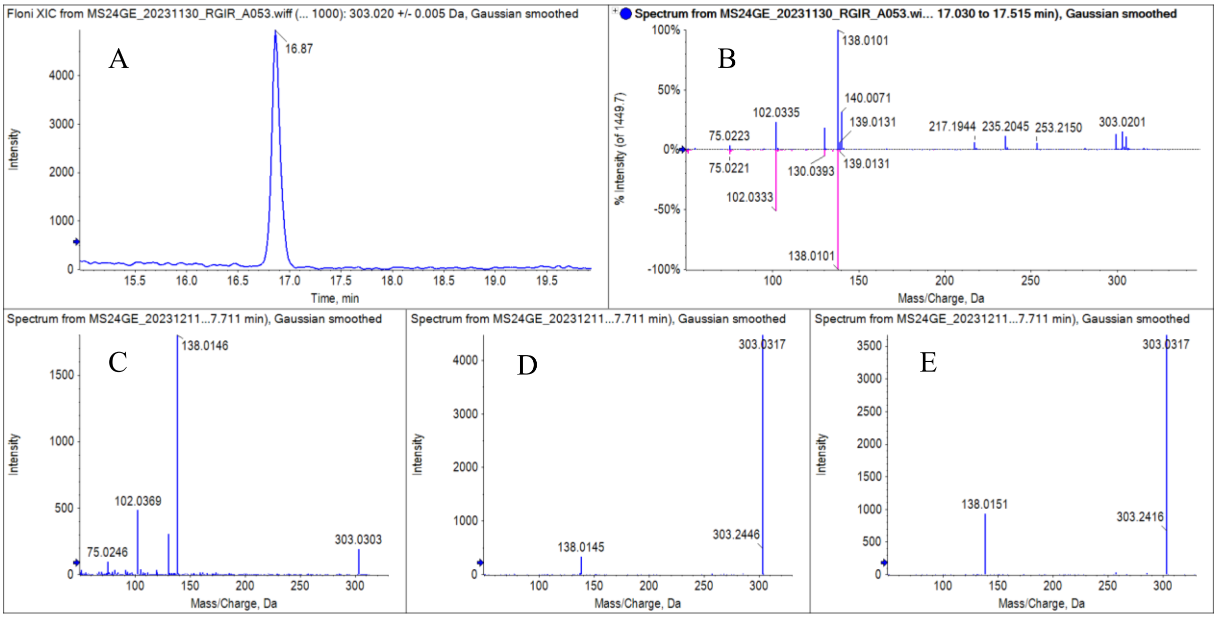

Figure S38. Detection of clofentezine in a white wine sample W18 A) TOF MS XIC of *m/z* 303.020 B) Upper trace CID SWATH/MS spectrum of peak at RT = 16.9 and lower trace SWATH MS/MS CID spectrum of clofentezine C) MRMHR CID D), MRMHR EAD, and E) MRMHR UVPD spectra of peak at RT = 16.9 in sample W18.

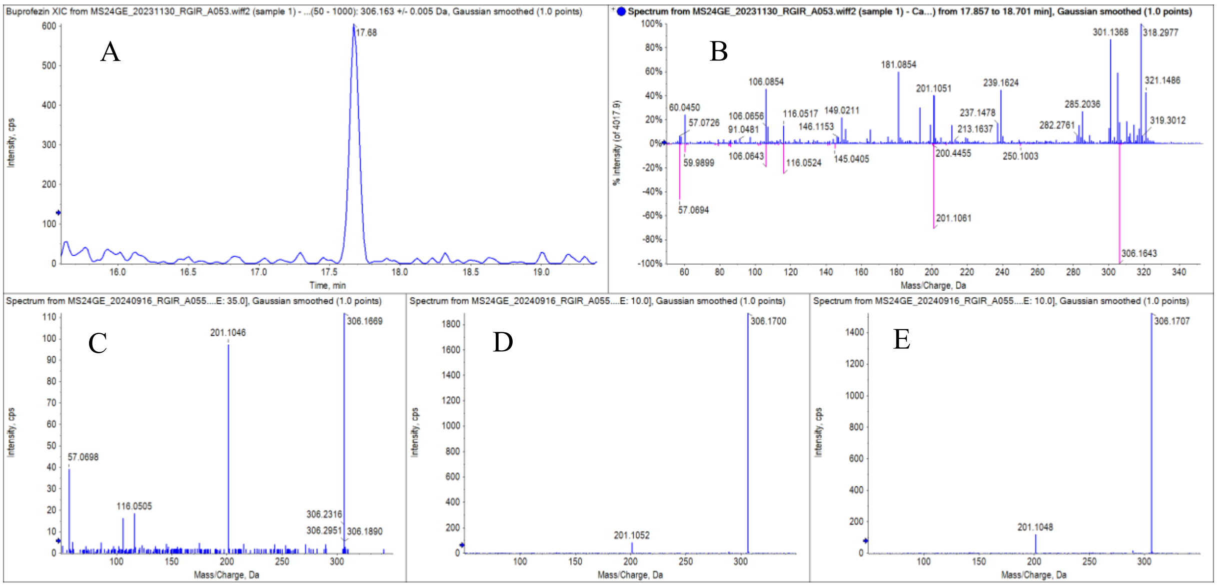

Figure S39. Detection of buprofezin in a white wine sample W18 A) TOF MS XIC of *m/z* 306.163 B) Upper trace CID SWATH/MS spectrum of peak at RT = 17.7 and lower trace SWATH MS/MS CID spectrum of buprofezin C) MRMHR CID D), MRMHR EAD, and E) MRMHR UVPD spectra of peak at RT = 17.7 in sample W18.

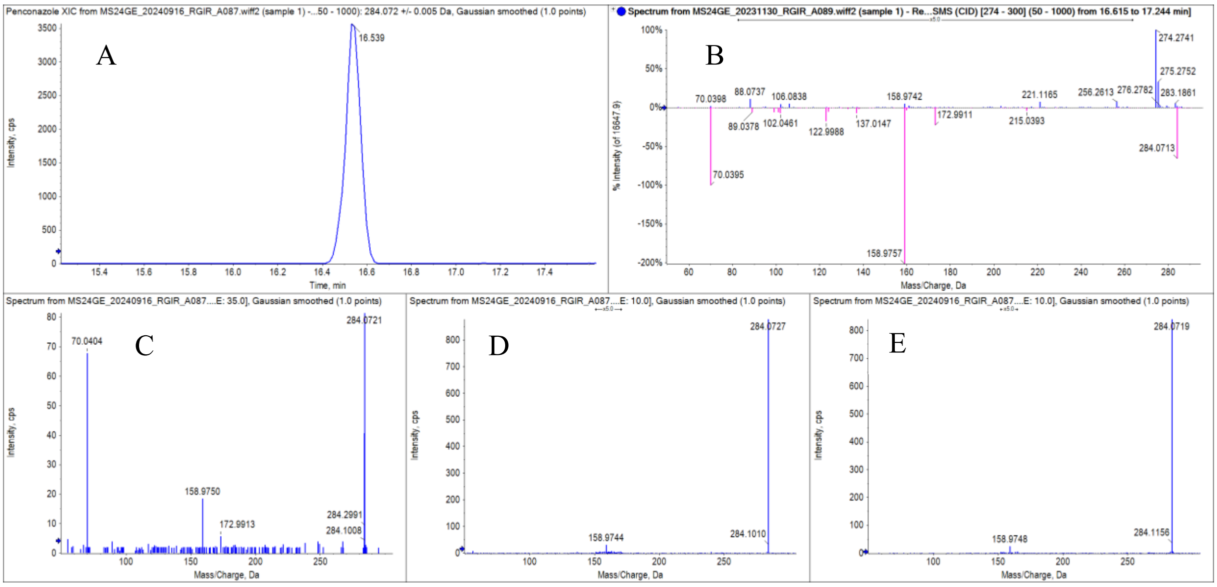

Figure S40. Detection of penconazole in a red wine sample W34 A) TOF MS XIC of *m/z* 284.072 B) Upper trace CID SWATH/MS spectrum of peak at RT = 16.5 and lower trace SWATH MS/MS CID spectrum of penconazole C) MRMHR CID D), MRMHR EAD, and E) MRMHR UVPD spectra of peak at RT = 16.5 in sample W34.

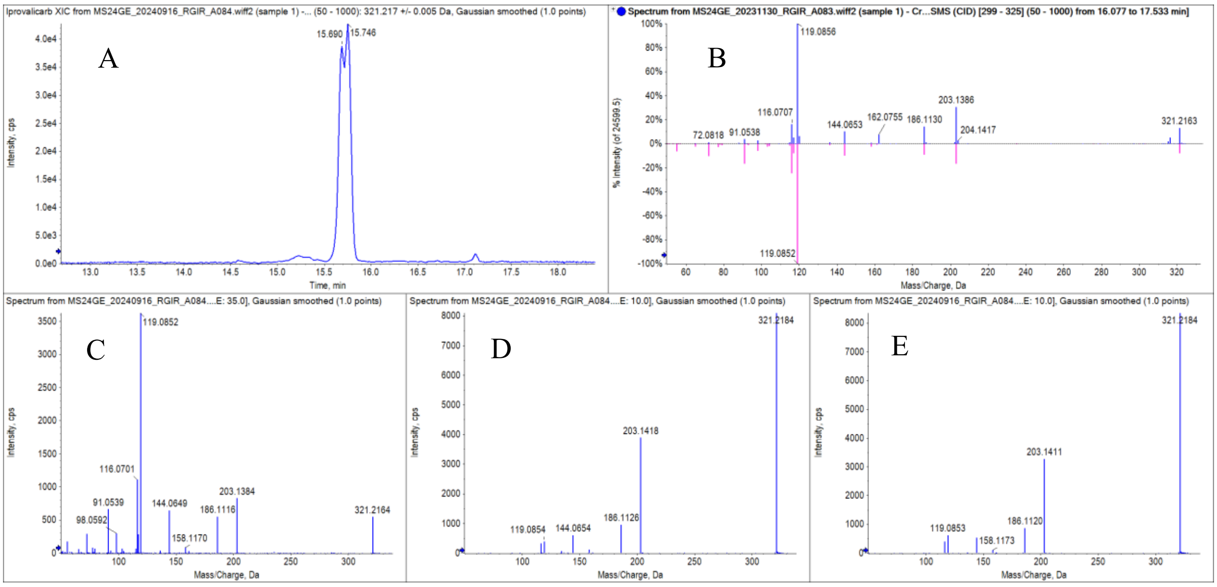

Figure S41. Detection of iprovalicarb in a red wine sample W13 A) TOF MS XIC of *m/z* 321.217 B) Upper trace CID SWATH/MS spectrum of peak at RT = 15.7 and lower trace SWATH MS/MS CID spectrum of penconazole C) MRMHR CID D), MRMHR EAD, and E) MRMHR UVPD spectra of peak at RT = 15.7 in sample W13.

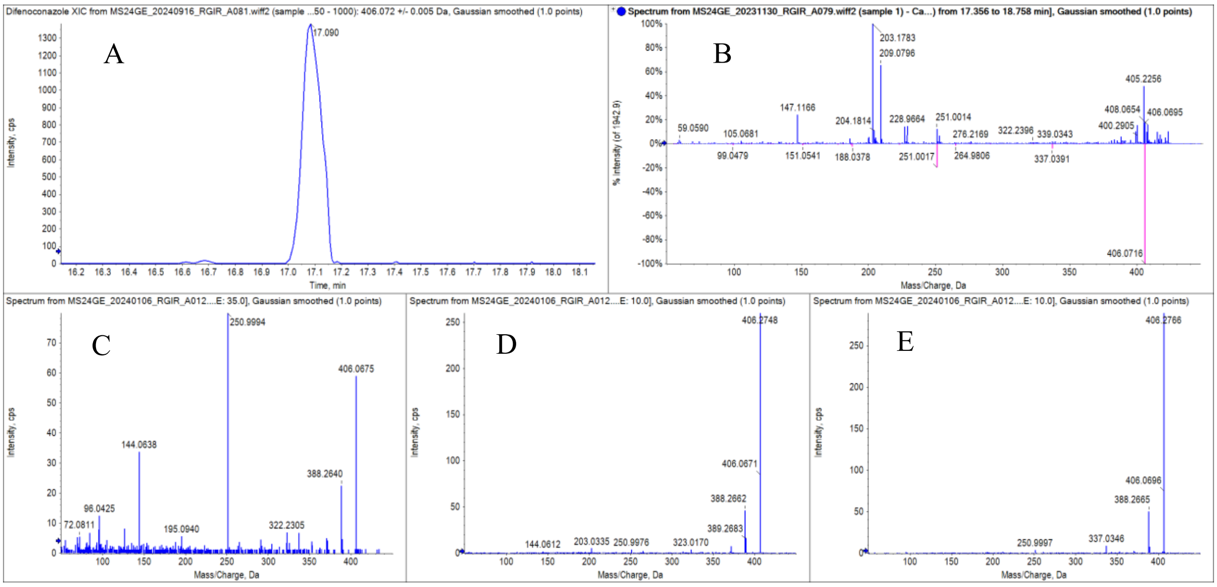

Figure S42. Detection of difenoconazole in red wine sample W17 A) TOF MS XIC of *m/z* 406.072 B) Upper trace CID SWATH/MS spectrum of peak at RT = 17.1 and lower trace SWATH MS/MS CID spectrum of difenoconazole C) MRMHR CID D), MRMHR EAD, and E) MRMHR UVPD spectra of peak at RT = 17.1 in sample W17.

.

Table S1. List of the 168 pesticides: CAS number, formula, exact mass Log P, retention time and observed fragment in CID/EAD/UVPD. [1] are the pesticides showing doubly charged radical cation. The MS/MS spectra of standard pesticides in MassBank format can be found at: 10.26037/yareta:idzwd42n4feaddt7fydqefavve

Pesticides	CAS	Formula	Exact Mass $[M+H]^+$ (*m/z*)	Log P	Retention time (min)	Fragmentation		
						CID	EAD	UVPD
3-Hydroxycarbofuran	16655-82-6	C12H15NO4	238.1074	1.65	9.44	x	x	x
Acetamiprid	135410-20-7	C10H11ClN4	223.0745	1.4	9.49	x	x	x
Acibenzolar-S-methyl	135158-54-2	C8H6N2OS2	210.9994	2.3	14.22	x	x	x
Alanycarb	83130-01-2	C17H25N3O4S2	400.1359	3.4	15.82	x	x	x
Aldicarb sulfone	1646-88-4	C7H14N2O4S	223.0747	-0.60	6.7	x	x	x
Aldicarb sulfoxide	1646-87-3	C7H14N2O3S	207.0798	-0.20	6.25	x	x	x
Ametryn	834-12-8	C9H17N5S	228.1277	3	14.25	x	x	x
Aminocarb	2032-59-9	C11H16N2O2	209.1285	1.90	11.85	x	x	x
Azoxystrobin	131860-33-8	C22H17N3O5	404.1241	3.7	14.06	x	x	x
Benalaxyl	71626-11-4	C20H23NO3	326.1751	3.4	16	x	x	x
Bendiocarb	22781-23-3	C11H13NO4	224.0917	1.7	11.9	x	x	x
Benfuracarb	82560-54-1	C20H30N2O5S	411.1948	4.2	17.04	x	x	x
Benzoximate	29104-30-1	C18H18ClNO5	364.0946	4.7	16.34	x	x	x
Bifenazate	149877-41-8	C17H20N2O3	301.1547	4.2	14.94	x	x	x
Bitertanol	55179-31-2	C20H23N3O2	338.1863	4.2	16.29	x	x	x
**Boscalid**[1]	188425-85-6	C18H12Cl2N2O	343.0399	4.9	14.43	x	x	x
Bromuconazole	116255-48-2	C13H12BrCl2N3O	375.9614	3.6	14,88 or 15,72	x	x	
Bupirimate	41483-43-6	C13H24N4O3S	317.1642	2.7	15.62	x	x	x
Buprofezin	69327-76-0	C16H23N3OS	306.1635	5.6	17.37	x	x	x
Carbaryl	63-25-2	C12H11NO2	202.0863	2.4	12.33	x	x	x
Carbendazim	10605-21-7	C9H9N3O2	192.0768	1.50	10.17	x	x	x
Carbetamide	16118-49-3	C12H16N2O3	237.1234	1.6	11.35	x	x	x
Carbofuran	1563-66-2	C12H15NO3	222.1125	2.3	11.91	x	x	x
Carboxin	5234-68-4	C12H13NO2S	236.0740	2.1	12.27	x	x	x
Carfentrazone-ethyl	128639-02-1	C15H14Cl2F3N3O3	412.0437	4	15.77	x	x	x
Chlorantraniliprole	500008-45-7	C18H14BrCl2N5O2	481.9781	4.8	13.76	x	x	x
**Chlorfluazuron**[1]	71422-67-8	C20H9Cl3F5N3O3	539.9702	6.7	18.13	x	x	x
**Chloroxuron**[1]	1982-47-4	C15H15ClN2O2	291.0895	3.5	14.96	x	x	x

Chlortoluron	15545-48-9	C10H13ClN2O	213.0789	2.4	12.9	x	x	x
Clethodim	99129-21-2	C17H26ClNO3S	360.1395	3.8	15.26	x	x	x
Clofentezine	74115-24-5	C14H8Cl2N4	303.0199	3.2	16.28	x	x	x
Clothianidin	210880-92-5	C6H8ClN5O2S	250.0160	1.3	8.92	x	x	x
Cyazofamid	120116-88-3	C13H13ClN4O2S	325.0521	2.6	15.34	x	x	x
Cycluron	2163-69-1	C11H22N2O	199.1805	2.4	13.48	x	x	x
Cymoxanil	57966-95-7	C7H10N4O3	199.0826	0.70	9.94	x	x	x
Cyproconazole	113096-99-4	C15H18ClN3O	292.1211	2.9	14.76	x	x	
Cyprodinil	121552-61-2	C14H15N3	226.1339	3.1	15.96	x	x	x
Cyromazine	66215-27-8	C6H10N6	167.1040	-0.2	5.42	x	x	x
Desmedipham	13684-56-5	C16H16N2O4	301.1183	3.4	13.64	x	x	x
Dicrotophos	141-66-2	C8H16NO5P	238.0839	0.0	8.49	x	x	x
Diethofencarb	87130-20-9	C14H21NO4	268.1543	2.8	14.14	x	x	x
**Difenoconazole**[1]	119446-68-3	C19H17Cl2N3O3	406.0720	4	16.5	x	x	x
Diflubenzuron	35367-38-5	C14H9ClF2N2O2	311.0393	3.9	15.52	x	x	x
Dimethoate	60-51-5	C5H12NO3PS2	230.0069	0.80	9.37	x	x	x
**Dimethomorph**[1]	110488-70-5	C21H22ClNO4	388.1310	3.9	14.62	x	x	x
Dimoxystrobin	149961-52-4	C19H22N2O3	327.1703	3.9	15.7	x	x	x
Diniconazole	83657-24-3	C15H17Cl2N3O	326.0821	4.2	16.54	x	x	x
Dinotefuran	165252-70-0	C7H14N4O3	203.1139	0	6.28	x	x	x
Dioxacarb	6988-21-2	C11H13NO4	224.0917	0.5	9.35	x	x	x
Diuron	330-54-1	C9H10Cl2N2O	233.0243	2.7	13.46	x	x	x
**Epoxiconazole**[1]	135319-73-2	C17H13ClFN3O	330.0804	3.2	15.28	x	x	x
Etaconazole	60207-93-4	C14H15Cl2N3O2	328.0614	3	15.21	x	x	
Ethiprole	181587-01-9	C13H9Cl2F3N4OS	396.9899	3.7	14.39	x	x	x
Ethirimol	23947-60-6	C11H19N3O	210.1601	1.7	13.42	x	x	x
Ethofumesate	26225-79-6	C13H18O5S	287.0948	2.7	14.19	x	x	x
**Etoxazole**[1]	153233-91-1	C21H23F2NO2	360.1770	5.4	17.86	x	x	x
Fenamidone	161326-34-7	C17H17N3OS	312.1165	4.1	14.34	x	x	x
Fenarimol	60168-88-9	C17H12Cl2N2O	331.0399	3.6	15.18	x	x	x

Fenazaquin	120928-09-8	C20H22N2O	307.1805	5.7	18.44	x	x	x
**Fenbuconazole**[1]	114369-43-6	C19H17ClN4	337.1215	3.2	15.42	x	x	x
Fenhexamid	126833-17-8	C14H17Cl2NO2	302.0709	4.4	15.1	x	x	x
Fenobucarb	3766-81-2	C12H17NO2	208.1332	2.8	14.56	x	x	x
Fenoxycarb	79127-80-3	C17H19NO4	302.1387	4.3	15.6	x	x	x
Fenpropimorph	67564-91-4	C20H33NO	304.2635	5.2	18.72	x	x	
Fenpyroximate	111812-58-9	C24H27N3O4	422.2074	4.9	18.05	x	x	x
Fenuron	101-42-8	C9H12N2O	165.1022	1	9.05	x	x	x
Flonicamid	158062-67-0	C9H6F3N3O	230.0536	0.8	7.42	x	x	x
Flubendiamide	272451-65-7	C23H22F7IN2O4S	683.0306	5.3	15.77	x	x	x
Flufenacet	142459-58-3	C14H13F4N3O2S	364.0737	3.6	15.23	x	x	x
**Flufenoxuron**[1]	101463-69-8	C21H11ClF6N2O3	489.0435	6.3	17.81	x	x	x
Fluometuron	2164-17-2	C10H11F3N2O	233.0896	2.4	12.78	x	x	x
Fluoxastrobin	361377-29-9	C21H16ClFN4O5	459.0866	5.1	15.02	x	x	x
Fluquinconazole	136426-54-5	C16H8Cl2FN5O	376.0163	4	15.04	x	x	x
Flusilazole	85509-19-9	C16H15F2N3Si	316.1076	3.84	15.55	x	x	x
Flutolanil	66332-96-5	C17H16F3NO2	324.1206	3.7	14.64	x	x	x
Flutriafol	76674-21-0	C16H13F2N3O	302.1099	2.3	13.15	x	x	x
**Forchlorfenuron**[1]	68157-60-8	C12H10ClN3O	248.0585	2.4	13.46	x	x	x
Fuberidazole	3878-19-1	C11H8N2O	185.0709	2.20	11.55	x	x	x
Furathiocarb	65907-30-4	C18H26N2O5S	383.1635	4.7	17.18	x	x	x
Hexaconazole	79983-71-4	C14H17Cl2N3O	314.0821	3.7	16.25	x	x	
Hexaflumuron	86479-06-3	C16H8Cl2F6N2O3	460.9889	5.7	16.76	x	x	x
Hexythiazox	78587-05-0	C17H21ClN2O2S	353.1085	4.5	17.66	x	x	x
Imazalil	35554-44-0	C14H14Cl2N2O	297.0556	3.8	15.81	x	x	x
Imidacloprid	138261-41-3	C9H10ClN5O2	256.0596	1.2	8.81	x	x	x
Indoxacarb	144171-61-9	C22H17ClF3N3O7	528.0780	4.8	16.65	x	x	x
**Ipconazole**[1]	125225-28-7	C18H24ClN3O	334.1681	4.1	16.83	x	x	
Iprovalicarb	140923-17-7	C18H28N2O3	321.2173	3.7	15.06	x	x	x
Isoprocarb	2631-40-5	C11H15NO2	194.1176	2.3	13.1	x	x	x

Isoproturon	34123-59-6	C12H18N2O	207.1492	2.9	13.3	x	x	x
Kresoxim-methyl	143390-89-0	C18H19NO4	314.1387	4.1	15.78	x	x	x
Linuron	330-55-2	C9H10Cl2N2O2	249.0192	3.2	14.21	x	x	x
Mandipropamid	374726-62-2	C23H22ClNO4	412.1310	3.9	14.49	x	x	x
Mefenacet	73250-68-7	C16H14N2O2S	299.0849	3.2	14.86	x	x	x
Mepanipyrim	110235-47-7	C14H13N3	224.1182	3.3	15.08	x	x	x
Mepronil	55814-41-0	C17H19NO2	270.1489	3.7	14.72	x	x	x
Mesotrione	104206-82-8	C14H13NO7S	340.0485	0.7	6.48	x	x	x
Metalaxyl	57837-19-1	C15H21NO4	280.1543	1.6	13.3	x	x	x
Metconazole	125116-23-6	C17H22ClN3O	320.1524	3.7	16.25	x	x	x
Methabenzthiazuron	18691-97-9	C10H11N3OS	222.0696	1.9	13.04	x	x	x
Methamidophos	10265-92-6	C2H8NO2PS	142.0086	-0.9	2.61	x	x	x
Methiocarb	2032-65-7	C11H15NO2S	226.0896	2.9	14.31	x	x	x
Methomyl	16752-77-5	C5H10N2O2S	163.0536	0.6	7.32	x	x	x
Methoprotryne	841-06-5	C11H21N5OS	272.1540	2.8	14.17	x	x	x
Methoxyfenozide	161050-58-4	C22H28N2O3	369.2173	4.6	14.74	x	x	x
**Metobromuron**[1]	3060-89-7	C9H11BrN2O2	259.0077	2.4	12.97	x	x	x
Metribuzin	21087-64-9	C8H14N4OS	215.0961	1.7	11.8	x	x	x
Mevinphos	7786-34-7	C7H13O6P	225.0523	1.2	10.34	x	x	x
Mexacarbate	315-18-4	C12H18N2O2	223.1441	2.5	14.76	x	x	x
Monocrotophos	6923-22-4	C7H14NO5P	224.0682	-0.2	8.07	x	x	x
Monolinuron	1746-81-2	C9H11ClN2O2	215.0582	2.3	12.55	x	x	x
**Moxidectin**[1]	113507-06-5	C37H53NO8	640.3844	4.3	13.3	x	x	
Myclobutanil	88671-89-0	C15H17ClN4	289.1215	2.9	14.84	x	x	x
Neburon	555-37-3	C12H16Cl2N2O	275.0712	3.8	15.76	x	x	
Nitenpyram	120738-89-8	C11H15ClN4O2	271.0956	2.4	6.99	x	x	x
Nuarimol	63284-71-9	C17H12ClFN2O	315.0695	2.2	14.22	x	x	x
Omethoate	1113-02-6	C5H12NO4PS	214.0297	-0.9	5.61	x	x	x
Oxadixyl	77732-09-3	C14H18N2O4	279.1339	1.8	11.17	x	x	x
Oxamyl	23135-22-0	C7H13N3O3S	220.0750	-0.5	9.45	x	x	x

Penconazole	66246-88-6	C13H15Cl2N3	284.0716	4,4	15.88	x	x	x
Pencycuron	66063-05-6	C19H21ClN2O	329.1415	4.8	16.5	x	x	x
Phenmedipham	13684-63-4	C16H16N2O4	301.1183	3.6	13.8	x	x	x
Picoxystrobin	117428-22-5	C18H16F3NO4	368.1104	3.6	15.6	x	x	x
Pirimicarb	23103-98-2	C11H18N4O2	239.1503	1.7	12.86	x	x	x
Prochloraz	67747-09-5	C15H16Cl3N3O2	376.0381	4.6	16.28	x	x	x
Promecarb	2631-37-0	C12H17NO2	208.1332	3.1	14.1	x	x	x
Prometon	1610-18-0	C10H19N5O	226.1662	3	14.05	x	x	x
Prometryne	7287-19-6	C10H19N5S	242.1434	3.5	15.2	x	x	x
Propamocarb	24579-73-5	C9H20N2O2	189.1598	1.2	6.3	x	x	x
Propiconazole	60207-90-1	C15H17Cl2N3O2	342.0771	3.5	16.11	x	x	x
Propoxur	114-26-1	C11H15NO3	210.1125	1.5	11.8	x	x	x
Pymetrozine	123312-89-0	C10H11N5O	218.1036	0	7.81	x	x	x
Pyracarbolid	24691-76-7	C13H15NO2	218.1176	2.1	12.07	x	x	x
Pyraclostrobin	175013-18-0	C19H18ClN3O4	388.1059	4.1	16.17	x	x	x
Pyridaben	96489-71-3	C19H25ClN2OS	365.1449	5.2	18.4	x	x	x
Pyrimethanil	53112-28-0	C12H13N3	200.1182	2.9	14.27	x	x	x
**Pyriproxyfen**[1]	95737-68-1	C20H19NO3	322.1438	4.8	17.48	x	x	x
**Quinoxyfen**[1]	124495-18-7	C15H8Cl2FNO	308.0040	5.1	17.6	x	x	x
**Rotenone**[1]	83-79-4	C23H22O6	395.1489	4.1	15.49	x	x	x
Secbumeton	26259-45-0	C10H19N5O	226.1662	3.2	13,96 or 14,37	x	x	x
Siduron	1982-49-6	C14H20N2O	233.1648	3.3	14.32	x	x	x
Simetryn	1014-70-6	C8H15N5S	214.1121	2.5	13.14	x	x	x
Spinetoram	187166-40-1	C42H69NO10	748.4994	5.9	18.3	x	x	
Spirodiclofen	148477-71-8	C21H24Cl2O4	411.1124	5.9	18.12	x	x	x
Spirotetramat	203313-25-1	C21H27NO5	374.1962	3.2	15.1	x	x	x
Spiroxamine	118134-30-8	C18H35NO2	298.2741	4.2	15.27	x	x	x
Sulfentrazone	122836-35-5	C11H10Cl2F2N4O3S	386.9892	2.3	12.2	x	x	
Tebuconazole	107534-96-3	C16H22ClN3O	308.1524	3.7	15.92	x	x	x
Tebufenozide	112410-23-8	C22H28N2O2	353.2224	3.9	15.63	x	x	x

Tebufenpyrad	119168-77-3	C18H24ClN3O	334.1681	4.5	17.24	x	x	x
Tebuthiuron	34014-18-1	C9H16N4OS	229.1118	1.6	12.15	x	x	x
Teflubenzuron	83121-18-0	C14H6Cl2F4N2O2	380.9815	4.9	17.4	x	x	x
**Temephos**[1]	3383-96-8	C16H20O6P2S3	466.9970	6	17.23	x	x	x
Terbumeton	33693-04-8	C10H19N5O	226.1662	3.1	13,96 or 14,37	x	x	x
Terbutryne	886-50-0	C10H19N5S	242.1434	3.7	15.36	x	x	x
**Tetraconazole**[1]	112281-77-3	C13H11Cl2F4N3O	372.0288	4.4	15.24	x	x	x
Thiabendazole	148-79-8	C10H7N3S	202.0433	2.5	11.19	x	x	x
Thiacloprid	111988-49-9	C10H9ClN4S	253.0309	2.2	10.21	x	x	x
Thiamethoxam	153719-23-4	C8H10ClN5O3S	292.0266	1.5	7.71	x	x	x
Thidiazuron	51707-55-2	C9H8N4OS	221.0492	1.3	11.96	x	x	x
Thiobencarb	28249-77-6	C12H16ClNOS	258.0714	3.4	16.41	x	x	x
Thiophanate-methyl	23564-05-8	C12H14N4O4S2	343.0529	2.6	11.78	x	x	x
Triadimefon	43121-43-3	C14H16ClN3O2	294.1004	2.8	14.78	x	x	x
Tricyclazole	41814-78-2	C9H7N3S	190.0433	1.7	10.55	x	x	x
Trifloxystrobin	141517-21-7	C20H19F3N2O4	409.1370	4.9	16.71	x	x	x
Triflumizole	68694-11-1	C15H15ClF3N3O	346.0929	4	16.9	x	x	x
Triflumuron	64628-44-0	C15H10ClF3N2O3	359.0405	5.6	16.27	x	x	x
Vamidothion	2275-23-2	C8H18NO4PS2	288.0488	0.3	9.4	x	x	x
Zoxamide	156052-68-5	C14H16Cl3NO2	336.0319	4.3	16.14	x	x	x
						168	168	158

Table S2. Description of fruit and vegetable juices analysed which were purchased in France and Switzerland. Pesticides found in these juices as well as their occurrences.

Sample	Juice composition	Fruits and vegetable origin	Origin	Pyrimethanil	Boscalid	Metalaxyl	Tebuconazole	Carbendazim	Cyprodinil	Pirimicarb	Acetamiprid	Imazalil	Azoxystrobin	Dimethomorph	Pyraclostrobin	Tebufenozide	Flonicamid	Fenhexamid	Mandipropamid	Methoxyfenozide	Trifloxystrobin	Chlorantraniliprole	Imidacloprid	Tetraconazole
J1	4.7% Orange, 4.4% lemon, 1.6% apple, 0.6% pineapple, 0.4% banana, 0.2% kiwi, 0.1% passion fruit	N/A	Germany	X								X												
J2	Apple/pear	Switzerland	Switzerland	X					X	X	X													
J3	50% Apple, 13% chokeberry, 10% acerola, 4% blackberry, 2% raspberry, lemon, strawberry, purple carrot, grape and green mate extract	N/A	Germany	X	X	X	X		X						X			X			X			
J4	49% Apple, 25% orange, 10% mango, acerola, 7% ginger, lemon, curcuma extract	N/A	Germany	None																				
J5	100% Orange	Mexico	Switzerland					X																
J6	100% Pomegranate	Turkey/Iran	Germany					X																
J7	White tea extract, 26% grape, 4% pomegranate, lemon	N/A	France		X	X							X											
J8	Apple / lemon	E.U.	France					X	X	X	X						X							
J9	Apple	N/A	France	X	X		X	X	X	X	X													
J10	Orange	N/A	France	X																				
J11	73% Orange, 22% coco water et mandarin	N/A	France	None																				

J12	18% Apple, 12% guava, 5% dragon fruits, lemon et beetroot	N/A	France	X		X				X		X	X											
J13	Pineapple	N/A	France	None																				
J14	Apple, orange, pineapple, mango, passion fruit, apricot, banana et guava	N/A	France	X				X				X												
J15	Pink grapefruit	N/A	France	X								X												
J16	Grape	N/A	France		X	X	X							X		X				X				
J17	Tomato	France	France											X					X					
J18	Carrot	France	France		X																			
J19	Apple	France	France	None																				
J20	Apple	UE	France	X	X		X				X											X	X	X
J21	Grape	UE	France		X	X	X					X												
Total				9	7	5	5	5	4	4	4	4	2	2	1	1	1	1	1	1	1	1	1	1

Table S3. Summary of red and white wines analysed with the pesticides detected and their occurrences.

Sample	Wine	Grape variety	Origin	Alcohol %	Dimethomorph	Pyrimethanil	Tebuconazole	Metalaxyl	Fenhexamid	Spiroxamine	Kresoxim-methyl	Zoxamide	Azoxystrobin	Carbendazim	Boscalid	Mandipropamid	Iprovalicarb	Tebufenozide	Chlorantraniliprole	Benalaxyl	Spinosad	Acetamiprid	Tetraconazole	Methoxyfenozide	Difenoconazole	Trifloxystrobin	Imidacloprid	Penconazole	Cyprodinil	Clofentezine	Buprofezin
W1	Red	Grenache noir, Syrah, Carignan	France	14.5	X					X		X				X										X					
W2	White	Pinot gris	France	N/A	X	X		X	X						X	X	X		X		X										
W3	Red	60% Tannat, 40% Cabernet Franc	France	N/A	X										X	X			X										X		
W4	Red	87% Tempranillo, 13% Garnacha	Spain	13.5	X	X	X	X		X	X		X	X	X	X	X	X		X			X	X							
W5	Red	50% Grenache, Mourvèdre	France	14.5	X			X		X											X										
W6	White	Grenache blanc, Rolle	France	13	X					X					X																
W7	Red	N/A	France	11	X		X	X					X		X			X		X											
W8	Red	Syrah, Grenache, Mourvèdre	France	14	X		X	X		X			X		X	X				X			X		X						
W9	Red	Syrah, Grenache, Carignan	France	14	X	X	X	X						X	X	X				X					X	X					
W10	White	Rolle, Roussanne Grenache blanc, Clairette	France	13	X	X	X						X			X															
W11	Red	Syrah, Grenache, Mourvèdre	France	14	X		X	X							X	X				X											
W12	Red	Loureiro, Trajadura, Vinhao	Portugal	9	X	X	X	X					X		X		X			X			X								
W13	White	Muscat blanc	Portugal	9	X	X	X	X					X	X	X		X		X	X		X									

W14	Red	Sangiovese	Italy	13	X	X		X							X					X			X	X							
W15	Red	Nero d'Avola	Italy	13	X	X		X						X	X		X	X						X							
W16	Red	Cabernet Sauvignon, Carménère	Chile	13	X	X	X						X		X									X							
W17	Red	Nielluccio, Sciaccarellum, Vermentino	France	12	X	X	X	X		X					X	X				X			X		X						
W18	White	Cabernet Sauvignon	U.S.A.	12.5			X						X	X	X				X				X	X						X	X
W19	White	Gewurztraminer	France	N/A	X	X	X	X	X	X	X	X																			
W20	White	Gewurztraminer	France	N/A	X	X	X		X	X	X	X																			
W21	White	Gewurztraminer	France	N/A	X	X	X	X	X	X	X	X	X	X																	
W22	White	Gewurztraminer	France	N/A	X	X	X	X	X	X		X																			
W23	White	Gewurztraminer	France	N/A	X	X	X	X	X		X																				
W24	White	Riesling	France	N/A	X	X	X	X	X	X	X	X	X																		
W25	White	Riesling	France	N/A	X	X	X	X	X	X	X	X																			
W26	White	Riesling	France	N/A	X	X	X	X	X	X	X			X																	
W27	White	Riesling	France	N/A	X	X	X	X	X	X	X	X	X																		
W28	White	Riesling	France	N/A	X	X	X	X	X		X	X					X	X													
W29	White	Pinot gris	France	N/A	X	X	X	X	X	X	X																				
W30	White	Pinot gris	France	N/A	X	X	X	X	X	X	X	X																			
W31	White	Pinot gris	France	N/A	X	X	X	X	X	X	X	X																			
W32	White	Pinot gris	France	N/A	X	X	X	X	X	X	X	X		X																	
W33	White	Pinot gris	France	N/A	X	X	X	X	X	X	X	X					X	X													
W34	Red	N/A	Italy	N/A	X			X						X														X			
W35	Red	N/A	Georgia (country)	N/A	X			X																			X				
W36	White	Gewurztraminer	France	13				X						X																	
W37	White	Sylvaner	France	12	X	X	X	X	X						X	X	X														
Total					35	26	27	30	17	19	15	13	11	10	16	10	8	5	4	9	2	1	6	5	3	2	1	1	1	1	1

Table S4. Concentration estimation of pesticides found in red and white wines, n.d. not detected.

				Pesticides concentration in red wines (pg/mL)		Pesticides concentration in white wines (pg/mL)	
**Pesticides**	**RT (min)**	***m/z***	**Formula**	**lower value**	**upper value**	**lower value**	**upper value**
Imidacloprid	9.53	256,0596	$C_9H_{10}ClN_5O_2$	12270		n.d.	
Acetamiprid	10.19	223,0745	$C_{10}H_{11}ClN_4$	n.d.		2791,0	
Carbendazim	10.86	192,0768	$C_9H_9N_3O_2$	3482	16931	284,5	22494
Metalaxyl	13.96	280,1543	$C_{15}H_{21}NO_4$	88,2	34513	68,5	6370
Chlorantraniliprole	14.38	481,9781	$C_{18}H_{14}BrCl_2N_5O_2$	1624	2522	520,3	1015
Azoxystrobin	14.69	404,1241	$C_{22}H_{17}N_3O_5$	130,7	944,5	102,9	349,4
Pyrimethanil	14.92	200,1182	$C_{12}H_{13}N_3$	201,3	1549	732,3	44974
Boscalid	15.07	343,0399	$C_{18}H_{12}Cl_2N_2O$	307,8	25831	331,0	5495
Mandipropamid	15.1	412,1310	$C_{23}H_{22}ClNO_4$	291,3	3020	658,4	5819
Dimethomorph	15.21	388,131	$C_{21}H_{22}ClNO_4$	398,9	16580	927,1	49925
Methoxyfenozide	15.36	369,2173	$C_{22}H_{28}N_2O_3$	1029	32803	n.d.	
Fenhexamid	15.70	302,0709	$C_{14}H_{17}Cl_2NO_2$	n.d.		629,3	39374
Iprovalicarb	15.70	321,2173	$C_{18}H_{28}N_2O_3$	781,0	5236	7803,1	7803
Tetraconazole	15.85	372,0288	$C_{13}H_{11}Cl_2F_4N_3O$	329,0	1229	n.d.	
Spiroxamine	16.01	298,2741	$C_{18}H_{35}NO_2$	20,2	404,1	108,9	2870
Tebufenozide	16.21	353,2224	$C_{22}H_{28}N_2O_2$	2380	3595	2691,4	4088
Kresoxim-methyl	16.36	314,1387	$C_{18}H_{19}NO_4$	403,6	403,6	197,3	4753
Penconazole	16.47	284,0716	$C_{13}H_{15}Cl_2N_3$	687,0	687,0	nd.	N.D.
Tebuconazole	16.52	308,1524	$C_{16}H_{22}ClN_3O$	248,6	3478	79,6	2652
Cyprodinil	16.52	226,1339	$C_{14}H_{15}N_3$	10630		N.D.	N.D.
Benalaxyl	16.6	326,1751	$C_{20}H_{23}NO_3$	179,9	1811	145,3	
Zoxamide	16.73	336,0319	$C_{14}H_{16}Cl_3NO_2$	191,0		156,1	1159
Clofentezine	16.87	303,0199	$C_{14}H_8Cl_2N_4$	2605		n.d.	
Difenoconazole	17.04	406,0720	$C_{19}H_{17}Cl_2N_3O_3$	238,1	454,9	n.d.	
Trifloxystrobin	17.17	409,1370	$C_{20}H_{19}F_3N_2O_4$	106,3	269,8	n.d.	
Buprofezin	17.68	306,1635	$C_{16}H_{23}N_3OS$	68,7		n.d.	
Spinosad	18.17	732,4681	$C_{41}H_{65}NO_{10}$	355,8		85,0	

Table S5. Summary of estimated lower limit of detection (LOD) for pesticides detected in juice, red, and white wine (n=27) based on the signal intensity of TOF MS, CID, EAD, and UVPD MS/MS, n.f. no fragment.

Pesticides	RT (min)	*m/z*	Formula	LOD (pg/mL)										
				TOF MS		CID			EAD			UVPD		
				Red wine	White wine	Fragments	Red wine	White wine	/z fragment	Red wine	White wine	n/z fragmen	Red wine	White wine
Imidacloprid	9.53	256.0596	$C_9H_{10}ClN_5O_2$	512.8	525.2	175.0975	135.0	153.7	175.0975	332.7	342.0	175.0975	50.0	36.5
Acetamiprid	10.19	223.0745	$C_{10}H_{11}ClN_4$	953.9	275.2	126.0100	893.7	27.0	126.0100	301.2	186.3	126.0100	71.7	42.4
Carbendazim	10.86	192.0768	$C_9H_9N_3O_2$	621.5	284.5	160.0505	160.9	42.0	160.0505	92.1	251.3	160.0505	155.6	47.9
Metalaxyl	13.96	280.1543	$C_{15}H_{21}NO_4$	64.5	59.5	220.1329	20.3	10.7	220.1329	17.4	22.4	220.1329	14.9	15.4
Chlorantraniliprole	14.38	481.9781	$C_{18}H_{14}BrCl_2N_5O_2$	28.7	22.0	283.9217	32.4	45.5	283.9217	103.7	41.9	283.9217	202.4	20.5
Azoxystrobin	14.69	404.1241	$C_{22}H_{17}N_3O_5$	19.6	54.3	372.097	3.3	1.5	372.097	7.3	25.8	372.097	4.8	12.9
Pyrimethanil	14.92	200.1182	$C_{12}H_{13}N_3$	12.7	9.5	107.0600	89.5	19.8	n.f.	-	-	n.f.	-	-
Boscalid	15.07	343.0399	$C_{18}H_{12}Cl_2N_2O$	91.7	18.0	139.9895	1110	713.2	139.9895	189.7	196.8	139.9895	313.4	111.2
Mandipropamid	15.1	412.1310	$C_{23}H_{22}ClNO_4$	23.8	31.8	328.1103	2.9	16.7	328.1103	19.2	28.3	328.1103	3.2	26.2
Dimethomorph	15.21	388.131	$C_{21}H_{22}ClNO_4$	31.4	72.4	301.0609	804.0	2329	301.0609	1478.3	582.1	301.0609	903.3	1098
Methoxyfenozide	15.36	369.2173	$C_{22}H_{28}N_2O_3$	1029.4	753.8	149.0595	5.5	13.7	149.0595	18.7	31.0	149.0595	11.8	24.6
Fenhexamid	15.70	302.0709	$C_{14}H_{17}Cl_2NO_2$	96.3	8.8	97.1010	267.7	30.6	176.9742	908.6	429.0	177.9817	900.6	579.2
Iprovalicarb	15.70	321.2173	$C_{18}H_{28}N_2O_3$	289.6	265.6	203.1383	115.8	80.5	203.1383	18.6	18.2	203.1383	9.5	18.8
Tetraconazole	15.85	372.0288	$C_{13}H_{11}Cl_2F_4N_3O$	18.8	20.6	158.9758	303.6	17.6	158.9758	493.7	298.7	158.9758	1950	3463
Spiroxamine	16.01	298.2741	$C_{18}H_{35}NO_2$	13.7	20.7	144.1371	1.7	1.1	n.f.	-	-	n.f	-	-
Tebufenozide	16.21	353.2224	$C_{22}H_{28}N_2O_2$	2380.4	1531.1	297.1594	31.6	32.6	297.1594	4.4	5.7	297.1594	4.8	6.5
Kresoxim-methyl	16.36	314.1387	$C_{18}H_{19}NO_4$	152.4	122.1	267.1008	38.2	86.3	267.1008	10.3	8.3	267.1008	10.3	8.7
Penconazole	16.47	284.0716	$C_{13}H_{15}Cl_2N_3$	11.0	8.7	158.9749	73.6	57.3	158.9749	116.2	113.0	158.9749	523.8	557.3
Tebuconazole	16.52	308.1524	$C_{16}H_{22}ClN_3O$	61.9	72.2	70.0399	190.0	33.8	n.f.	-	-	n.f	-	-
Cyprodinil	16.52	226.1339	$C_{14}H_{15}N_3$	12.9	8.6	n.f	-	n.f	n.f.	-	-	n.f	-	-
Benalaxyl	16.6	326.1751	$C_{20}H_{23}NO_3$	54.1	111.5	148.1122	2.7	4.7	148.1122	45.2	30.0	148.1122	51.8	29.7
Zoxamide	16.73	336.0319	$C_{14}H_{16}Cl_3NO_2$	7.1	5.0	186.9706	104.1	40.4	186.9706	252.4	55.6	186.9706	122.0	51.7
Clofentezine	16.87	303.0199	$C_{14}H_8Cl_2N_4$	299.1	213.6	138.0108	32.7	1.6	138.0108	25.0	15.3	138.0108	37.0	20.0
Difenoconazole	17.04	406.0720	$C_{19}H_{17}Cl_2N_3O_3$	36.2	28.8	251.0022	126.1	19.0	251.0022	427.9	155.3	251.0022	776.0	776.9
Trifloxystrobin	17.17	409.1370	$C_{20}H_{19}F_3N_2O_4$	5.2	1.3	186.0531	2.7	2.5	186.0531	62.5	41.4	186.0531	204.8	9.5
Buprofezin	17.68	306.1635	$C_{16}H_{23}N_3OS$	29.9	36.4	201.1056	11.4	1.8	201.1056	17.5	5.8	201.1056	16.6	6.6
Spinosad	18.17	732.4681	$C_{41}H_{65}NO_{10}$	59.7	45.5	142.1226	774.1	70.4	142.1226	571.2	690.7	142.1226	1689	1111
			**min**	**5.2**	**1.3**		**1.71**	**1.1**		**4.36**	**5.7**		**3.25**	6.5
			**max**	**2380**	**1531**		**1110**	**2329**		**1478**	**690.7**		**1950**	3463
